# The Mitotic Checkpoint Complex Requires an Evolutionary Conserved Cassette to Bind and Inhibit Active APC/C

**DOI:** 10.1016/j.molcel.2016.11.006

**Published:** 2016-12-15

**Authors:** Barbara Di Fiore, Claudia Wurzenberger, Norman E. Davey, Jonathon Pines

**Affiliations:** 1 The Gurdon Institute and Department of Zoology, University of Cambridge, Cambridge CB2 1QN, UK; 2 Conway Institute of Biomolecular and Biomedical Sciences, University College Dublin, Dublin 4, Ireland; 3 Division of Cancer Biology, The Institute of Cancer Research, 237 Fulham Road, London SW3 6JB, UK

**Keywords:** mitosis, checkpoint, Spindle Assembly Checkpoint, Anaphase Promoting Complex, Cyclosome, ABBA motif, BubR1, Cdc20

## Abstract

The Spindle Assembly Checkpoint (SAC) ensures genomic stability by preventing sister chromatid separation until all chromosomes are attached to the spindle. It catalyzes the production of the Mitotic Checkpoint Complex (MCC), which inhibits Cdc20 to inactivate the Anaphase Promoting Complex/Cyclosome (APC/C). Here we show that two Cdc20-binding motifs in BubR1 of the recently identified ABBA motif class are crucial for the MCC to recognize active APC/C-Cdc20. Mutating these motifs eliminates MCC binding to the APC/C, thereby abolishing the SAC and preventing cells from arresting in response to microtubule poisons. These ABBA motifs flank a KEN box to form a cassette that is highly conserved through evolution, both in the arrangement and spacing of the ABBA-KEN-ABBA motifs, and association with the amino-terminal KEN box required to form the MCC. We propose that the ABBA-KEN-ABBA cassette holds the MCC onto the APC/C by binding the two Cdc20 molecules in the MCC-APC/C complex.

## Highlights

• The N-terminal half of BubR1 contains two ABBA motifs that bind Cdc20
• The motifs are required for the MCC to bind and inhibit active APC/C-Cdc20
• These ABBA motifs are essential for the spindle assembly checkpoint
• The ABBA motifs flank a KEN box to form a cassette highly conserved in evolution

## Graphical Abstract

**Figure:**
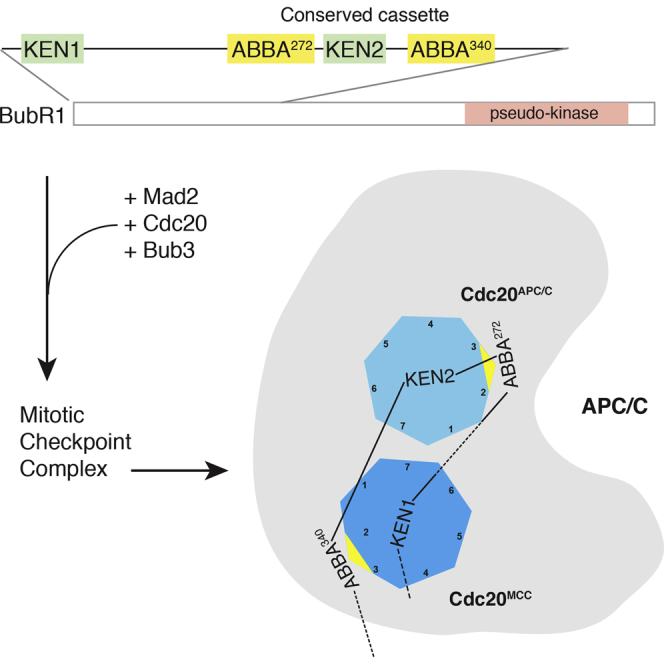

## Teaser

Di Fiore et al. show that two ABBA motifs in BubR1 are crucial for the Spindle Assembly Checkpoint because they are needed for the Mitotic Checkpoint Complex (MCC) to bind and inhibit the Anaphase Promoting Complex/Cyclosome (APC/C). Their results show how the MCC inhibits active APC/C.

## Introduction

Genomic stability in mitosis is ensured by the Spindle Assembly Checkpoint (SAC), which monitors proper chromosome attachment to the mitotic spindle. The SAC works through unattached kinetochores catalyzing the production of the Mitotic Checkpoint Complex (MCC) (Hardwick et al., 2000, Sudakin et al., 2001), which inhibits Cdc20 to prevent the Anaphase Promoting Complex/Cyclosome (APC/C) ubiquitin ligase from recognizing securin and Cyclin B1 (Fang et al., 1998, Hwang et al., 1998, Kim et al., 1998, Primorac and Musacchio, 2013). According to the accepted “Mad2 Template” model (De Antoni et al., 2005), Mad1-Mad2 heterodimers on unattached kinetochores catalyze a conformational change in a second Mad2 protein that allows it to bind Cdc20 (Luo et al., 2002, Sironi et al., 2002), and this Mad2-Cdc20 complex binds BubR1-Bub3 to form the hetero-tetrameric MCC (Chao et al., 2012, Herzog et al., 2009, Sudakin et al., 2001). Mad2 in the MCC prevents Cdc20 from binding and activating the APC/C (Izawa and Pines, 2012, Zhang and Lees, 2001) and the MCC itself acts as a pseudo-substrate inhibitor (Burton and Solomon, 2007, Chao et al., 2012, Lara-Gonzalez et al., 2011). Cdc20 binding to Mad2 is mutually exclusive with binding to the APC/C (Izawa and Pines, 2012); yet, we and others showed that, even after the APC/C is activated by binding Cdc20, the SAC can rapidly inhibit APC/C-Cdc20 should kinetochore-microtubule attachment be perturbed (Clute and Pines, 1999, Dick and Gerlich, 2013). How the SAC could inhibit the active APC/C-Cdc20 complex was unclear, but it was hypothesized (Burton and Solomon, 2007, Primorac and Musacchio, 2013) and recently shown (Izawa and Pines, 2015) that the MCC can recognize a second Cdc20 molecule through its D-box and KEN box degron receptor sites (Izawa and Pines, 2015).

Although the MCC only binds weakly to Cdc20 (Izawa and Pines, 2015), it binds to APC/C-Cdc20 with sufficient affinity to be co-purified by gel filtration or immunoprecipitation (Herzog et al., 2009, Morrow et al., 2005, Nilsson et al., 2008, Sudakin et al., 2001). How the MCC binds stably to the APC/C is not known: it must involve APC/C-bound Cdc20 because mutating the latter’s isoleucine arginine (IR) tail destabilizes interaction with the MCC (Hein and Nilsson, 2014), but mutating the pseudo-substrate D-box and KEN box sites on BubR1 does not prevent MCC binding (Izawa and Pines, 2015). Thus, additional sites of interaction between the MCC and APC/C-Cdc20 must exist.

We and others recently described a conserved motif in the C-terminal half of BubR1 starting at residue 528 that binds to Cdc20 and is required for BubR1 to recruit Cdc20 to kinetochores (Di Fiore et al., 2015, Han et al., 2014, Lischetti et al., 2014). We named this the ABBA motif because it is conserved in Cyclin A, Bub1, BubR1, and Acm1 (Di Fiore et al., 2015), although it has also been called the Phe box in BubR1 (Díaz-Martínez et al., 2015). (Note that this was originally called the A motif in yeast Acm1 (Burton et al., 2011), where it binds to Cdh1 into a pocket that closely resembles the sequence in human Cdc20. See Supplemental Experimental Procedures and Figures S3A–S3C.) But we found that the ABBA^528^ motif in BubR1 plays only a minor role in the checkpoint (Di Fiore et al., 2015).

Here we report the identification of two ABBA motifs in the N-terminal half of BubR1. We show that these motifs flank a KEN box to form a cassette that is highly conserved through evolution and is essential for the SAC because it is required for the MCC to bind and inhibit APC/C-Cdc20. Our results help to explain how the MCC rapidly inactivates the active APC/C in mitosis.

## Results

### Two Additional BubR1 ABBA Motifs Bind Cdc20

**Figure 1:**
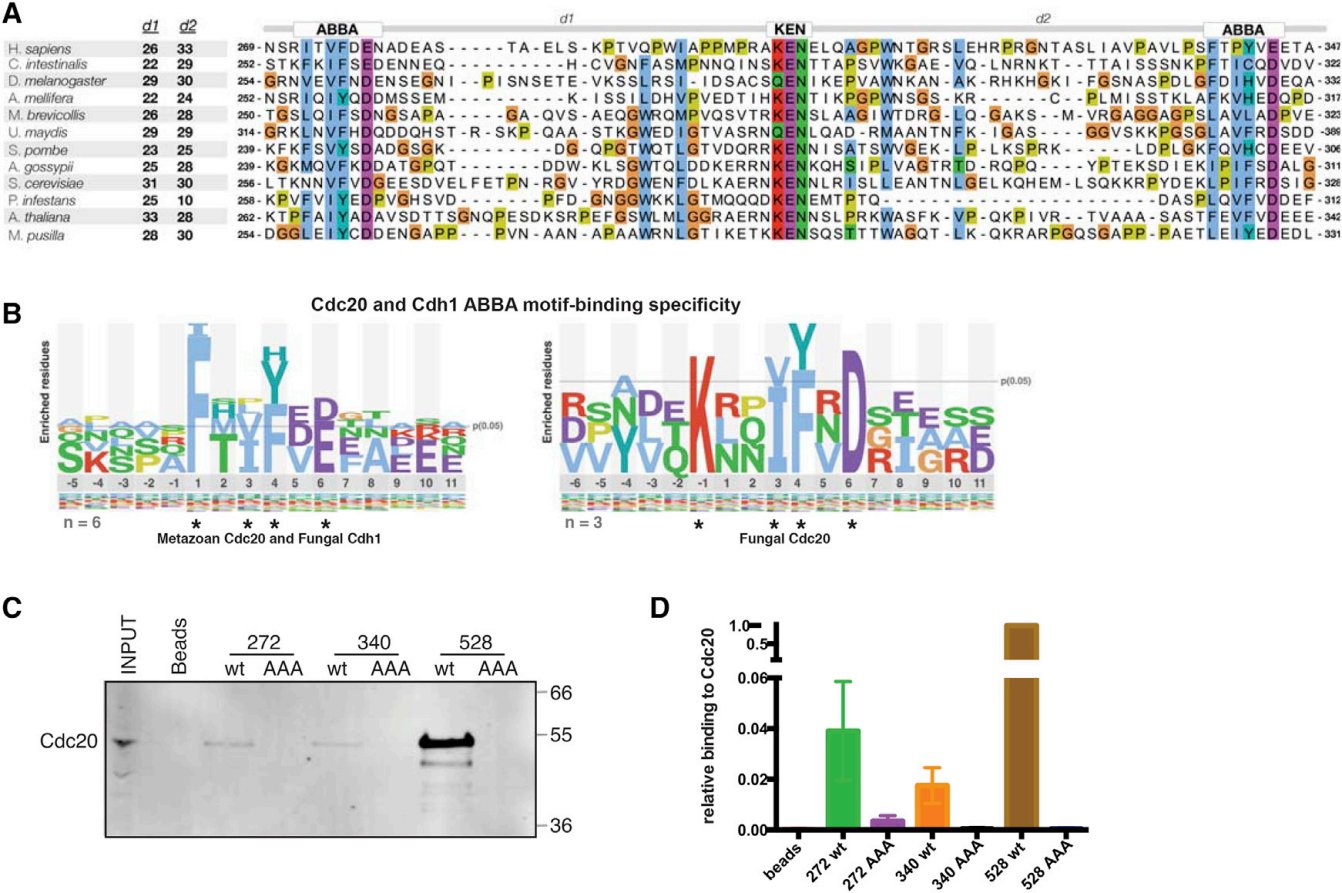
BubR1 ABBA Motifs Bind Cdc20 (A) Alignment of the central region of the MAD3/BUBR1-like proteins showing an ABBA-KEN-ABBA cassette conserved across the majority of eukaryotic kingdoms. The inter-motif spacing d1 (ABBA^272^-KEN) and d2 (KEN-ABBA^340^) are also shown. (B) Relative binomial logos created from the experimentally characterized ABBA motif instances and their flanking residues. Logos show the log^−10^ of the binomial probability (see Supplemental Experimental Procedures for details). Asterisks signify positions that show strong preferences for a particular amino acid or chemically similar grouping of amino acids (e.g., hydrophobic, aromatic, or acidic positions). Logos are split to emphasize the divergence of the specificity of the fungal Cdc20 from the metazoan Cdc20 and fungal Cdh1. See Supplemental Experimental Procedures for details. (C) Biotin-labeled peptides encompassing the BubR1 ABBA motifs were incubated with extract from HeLa cells synchronized in metaphase. Peptides in which positions 3, 4, and 6 of the consensus ABBA motif were mutated to alanine (AAA) were included as controls. (D) Cdc20 binding shown in (C) was quantified and the mean and SEM from three independent experiments are shown. See also Figure S1.

The original metazoan ABBA motif instances matched a consensus of Fx[ILV][FHY]x[DE] (Di Fiore et al., 2015). However, we noticed that there were two highly conserved, but non-canonical, ABBA motifs in the N-terminal half of BubR1 (Figure 1A), starting at residues 272 and 340 (see Supplemental Experimental Procedures for the bioinformatics analysis). These instances, one of which does not have a consensus phenylalanine residue (Figure 1A), indicate that the motif consensus is more degenerate than previously thought (Figure 1B); therefore, we prefer the “ABBA motif” name over “Phe box.” Peptides of ABBA^272^ and ABBA^340^ motifs could bind Cdc20 in metaphase HeLa cell extracts (Figures 1C and 1D), although more weakly than the ABBA^528^ peptide (Figure 1C), perhaps as a result of their non-canonical sequences. Control peptides in which alanine was substituted at conserved positions 3, 4, and 6 of the motif did not bind Cdc20. All three ABBA motifs appear to bind to the same site on Cdc20 because mutating residues in the ABBA binding pocket between blades 2 and 3 of the WD40 domain—either Y179E/I280Q (Di Fiore et al., 2015) or R262S (Díaz-Martínez et al., 2015)—reduced binding to all three peptides (Figures S1A and S1B).

### ABBA Motifs Are Required for the MCC to Bind and Inhibit APC/C-Cdc20

**Figure 2:**
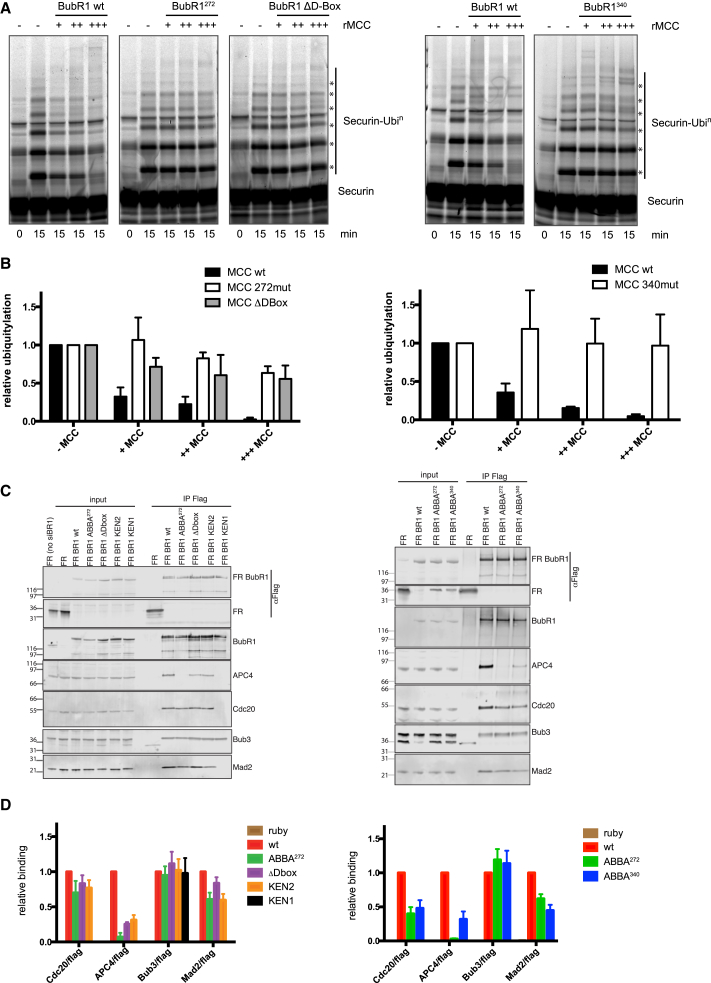
BubR1 ABBA^272^ and ABBA^340^ Are Essential for the MCC to Bind and Inhibit the APC/C (A) Apo-APC/C immunoprecipitated from Cdc20-depleted mitotic HeLa cell extracts was incubated with recombinant SBP-Cdc20 and increasing amounts of recombinant MCC (rMCC) containing wild-type, ABBA^272^ mutant, or ΔD-box mutant of BubR1 (left) or WT or ABBA^340^ mutant (right). Results shown are representative of three independent experiments. (B) Ubiquitylation assays shown in (A) were quantified as shown in Figure S1D. Mean ± SD of three independent experiments is shown. Ratios between Cdc20 (MCC) and SBP-Cdc20 added to the reaction are quantified in Figures S1E and S1F. (C) HeLa FRT/TO cell lines stably expressing inducible siRNA-resistant, FLAG-mRuby (FR)-BubR1 WT or mutants were transfected with siRNA to deplete endogenous BubR1. Anti-FLAG immunoprecipitations from nocodazole-arrested cells were analyzed by immunoblotting and visualized on a LI-COR Odyssey scanner. (D) Mean ± SEM of protein levels of the experiments shown in (C) from four (left graph) or three (right graph) independent experiments. See also Figure S1.

Although the C-terminal ABBA^528^ motif is not required to form the MCC (Di Fiore et al., 2015, Díaz-Martínez et al., 2015, Kaisari et al., 2016), since ABBA^272^ and ABBA^340^ are in the N-terminal half of BubR1, we asked whether they are required to form a functional MCC. We used baculovirus-infected Sf9 cells to co-express His_6_-Mad2, Cdc20, and Bub3 with streptavidin binding protein (SBP)-tagged BubR1 carrying inactivating mutations in either the ABBA^272^ or the ABBA^340^ motif. Both mutants were incorporated into recombinant MCC (rMCC) in the same manner as wild-type (WT) BubR1 (Figure S1C), but when we assayed the ability of the rMCC to inhibit APC/C-Cdc20, neither mutant form of rMCC could inhibit active APC/C in an in vitro ubiquitylation assay (Figures 2A and 2B). In this, they strongly resembled the BubR1 D-box mutant that cannot act as a pseudo-substrate inhibitor (Izawa and Pines, 2015; Figures 2A and 2B); therefore, we assayed whether the ABBA^272^ and ABBA^340^ motifs were needed for the MCC to recognize APC/C-Cdc20.

**Figure 3:**
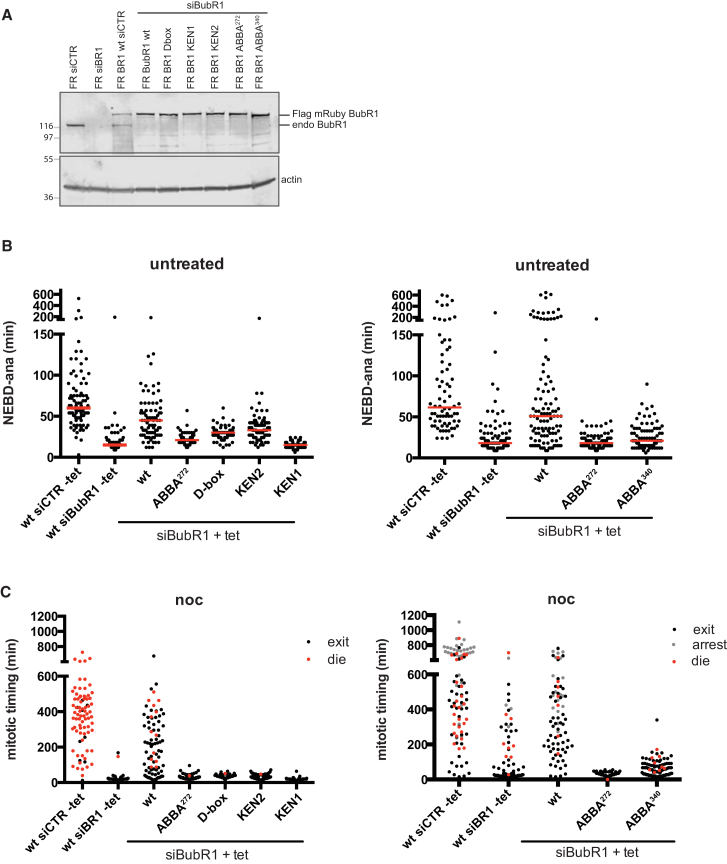
BubR1 ABBA^272^ and ABBA^340^ Are Essential for the SAC (A) HeLa FRT/TO cell lines stably expressing inducible siRNA-resistant, FLAG-mRuby-tagged, wild-type or mutant BubR1 were transfected with control siRNA (siCTR) or siRNA against BubR1. Immunoblotting analysis shows the relative expression levels and the efficiency of depletion. Actin is a loading control. (B) Mitotic timing of HeLa FRT/TO cell lines in (A) was measured with or without addition of tetracycline (tet). At least 80 cells per condition were analyzed. Results representative of three independent experiments. (C) Experiments were performed as in (B) except that nocodazole (0.33 μM) was added at the beginning of the filming. At least 80 cells per condition were analyzed. Results representative of three independent experiments. See also Figure S2.

We generated stable cell lines expressing small interfering RNA (siRNA)-resistant forms of FLAG-mRuby-tagged wild-type BubR1 or the ABBA^272^ or ABBA^340^ mutants from a tetracycline-inducible promoter. We depleted endogenous BubR1 and induced expression of the BubR1-ABBA mutants to physiological levels (Figure 3A). As controls, we assayed stable cell lines expressing BubR1 with inactivating mutations in the N-terminal KEN1 box at position 26, through which BubR1 binds Cdc20 within the core MCC (Burton and Solomon, 2007, Chao et al., 2012, King et al., 2007), or the D-box at position 224 or the KEN2 box at position 305 (between ABBA^272^ and ABBA^340^ motifs) that we previously showed are required to bind a second molecule of Cdc20 (Izawa and Pines, 2015). After enriching cells in prometaphase, we immunoprecipitated BubR1 and probed for MCC and APC/C components (Figures 2C and 2D; Figures S1G and S1H). As expected, the KEN1 mutant could not form the MCC, whereas the D-box, KEN2, ABBA^272^, and ABBA^340^ mutants could all be incorporated into the core MCC, although the levels of Mad2 co-immunoprecipitating with these mutants were reduced compared to wild-type, potentially indicating a difference in the efficiency of MCC assembly or stability (Figures 2C and 2D). The most striking difference, however, was in the amount of co-immunoprecipitated APC/C: the D-box, KEN2, and ABBA^340^ mutations markedly reduced the level of APC/C compared to wild-type, and APC/C binding was eliminated for the ABBA^272^ mutant (Figures 2C and 2D). We conclude that ABBA^272^ in particular is essential for the MCC to bind to the APC/C.

### ABBA Motifs Are Essential to Maintain the SAC In Vivo

We predicted that the ABBA^272^ and ABBA^340^ motifs would both be important for the SAC since they perturb MCC binding to the APC/C. To test this, we depleted endogenous BubR1 by siRNA and compared the ability of the D-box, KEN1, KEN2, ABBA^272^, and ABBA^340^ mutants to wild-type BubR1 to restore the SAC in two ways: by measuring the time taken from nuclear envelope breakdown (NEBD) to anaphase—which is set by the SAC—and by measuring how long cells could remain arrested in mitosis in the presence of spindle poisons. In both assays, the KEN1 mutant was the most impaired since the MCC cannot form, but the ABBA^272^ mutant was also profoundly impaired, to a greater extent even than the D-box and KEN2 mutants (Figures 3B and 3C). The ABBA^340^ mutant had a slightly less severe phenotype, but cells were still accelerated through prometaphase and unable to sustain a mitotic arrest in the presence of nocodazole (Figures 3B and 3C). Similar results were observed in the RPE1-hTERT untransformed cell line, where the ABBA^272^ mutant also showed a more severe phenotype than ABBA^340^ (Figure S2A). Note that unlike the ABBA^528^ motif, the ABBA^272^ and ABBA^340^ motifs were not required to recruit Cdc20 to kinetochores (Figures S2B and S2C). In agreement with these results, the Cdc20 mutants that were unable to bind the ABBA motifs were also unable to maintain a mitotic arrest (Figures S2D and S2E) in HeLa cells. Thus, we conclude that the ABBA^272^ and ABBA^340^ motifs are essential for the SAC by mediating MCC binding and inhibition of the APC/C.

### ABBA Motifs Are Part of a Conserved Cassette Important for the SAC

**Figure 4:**
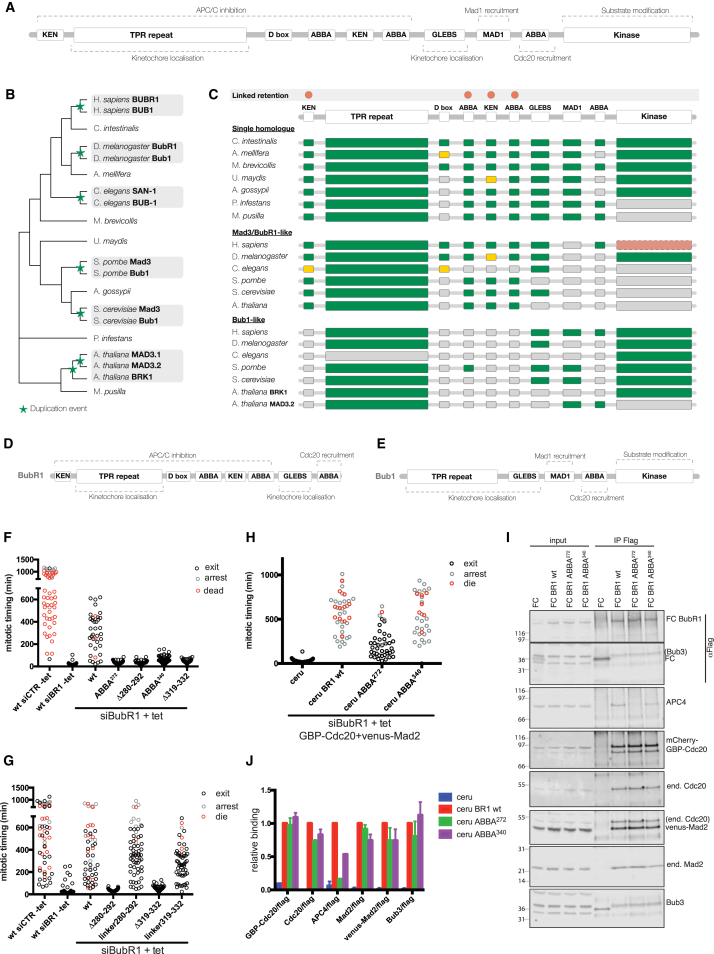
The ABBA^272^-KEN2-ABBA^340^ Module Is Conserved through Evolution (A) Predicted pre-duplication architecture of the ancestral protein to the human Bub1-like proteins—BubR1 and Bub1—based on *Ciona intestinalis* that has a single Bub1-like protein. The ancestral Bub1-like protein likely consisted of: an N-terminal KEN box pseudo-substrate domain; a TPR domain that stabilized the interaction between the N-terminal KEN and Cdc20 in the MCC (Chao et al., 2012) and bound to KNL1 to promote kinetochore recruitment (Krenn et al., 2012); a Bub3-binding motif (Wang et al., 2001); a MAD1 recruitment module (Klebig et al., 2009); an ABBA motif that promoted Cdc20 kinetochore recruitment (Di Fiore et al., 2015); and a C-terminal kinase domain (Bolanos-Garcia and Blundell, 2011, Suijkerbuijk et al., 2012). The region between the TPR domain and GLEBS motif also contained the putative ABBA motifs, a KEN box, and a D-box. (B) An evolutionary tree of the Bub1-like proteins in selected eukaryotic species. The tree contains two distinct classes of species: (1) those where a duplication event has resulted in two or more Bub1-like proteins (light gray boxes) and (2) those where a single Bub1-like protein is present. The position of these classes in the tree was chosen to define points of Bub1-like protein duplication (green star). (C) The modular architecture of the species in (B) grouped by (1) Bub1-like proteins from single homolog species, (2) BubR1-like proteins from multiple homolog species, and (3) Bub1-like proteins from multiple homolog species. Architecture shows the retained modules (green), inconclusively retained modules (yellow), retained modules characterized as non-functional (red), and potentially absent modules (gray). The modules that, post-duplication, have been simultaneously retained in BubR1 and lost in Bub1 independently on multiple occasions are marked above the architecture by red circles. Search details are shown in Table S1. (D and E) Architecture of the functional modules in the human BubR1 (D) and human Bub1 (E) showing the role of the retained modules post-subfunctionalization. See Figure S3 and http://slim.ucd.ie/abbakenabba/ for alignments. (F) HeLa FRT/TO cell lines stably expressing siRNA-resistant, FLAG-mRuby-tagged, WT or mutant BubR1 from a tetracycline (tet)-inducible promoter were transfected with control siRNA or siRNA against BubR1 (siBubR1), and nocodazole was added at the beginning of filming. Mitotic timing was measured with or without addition of tetracycline. The mitotic timing of WT BubR1 and ABBA^272^ or ABBA^340^ mutants was compared to deletion mutants in the inter-motif region. At least 50 cells per condition were analyzed. Results representative of three independent experiments. (G) HeLa FRT/TO cell lines expressing the indicated BubR1 proteins were treated as in (F) and the mitotic timings compared. At least 50 cells per condition were analyzed. Results representative of three independent experiments. (H) HeLa FRT/TO cell lines stably expressing siRNA-resistant, FLAG-Cerulean (FC) BubR1 WT or mutant from a tetracycline-inducible promoter were transfected with siRNA against BubR1 together with plasmids expressing mCherry-GBP-Cdc20 and Venus-Mad2. Mitotic timing was measured after addition of tetracycline. At least 35 cells per condition were analyzed. Results are representative of three independent experiments. (I) HeLa FRT/TO cell lines stably expressing inducible siRNA-resistant, FLAG-Cerulean-BubR1 WT or mutants were transfected as for (H), and anti-FLAG immunoprecipitations from nocodazole-arrested cells were analyzed by immunoblotting and visualized on a LI-COR Odyssey scanner. (J) The mean and SEM of protein levels from two independent experiments in (I). A third experiment was consistent with these results, but the siRNA did not deplete endogenous BubR1 to the same extent so these results were not included in the calculations.

Evolutionary analysis revealed a further aspect of the importance of ABBA^272^ and ABBA^340^ for the function of BubR1. ABBA^272^ and ABBA^340^ flank the similarly highly conserved KEN2 box that is required for the pseudo-substrate inhibitor role of BubR1 (Izawa and Pines, 2015). This ABBA-KEN-ABBA cassette is conserved across almost all major eukaryotic supergroups (including Opisthokonta, Amoebozoa, Plantae, Chromalveolata, and Excavata) (Figure 4A). The conservation of the cassette for over a billion years of evolution points to an important functional role—especially given the relative transience of most short linear motifs over long evolutionary timescales (Davey et al., 2012). Furthermore, the ABBA-KEN-ABBA cassette is always found associated with the N-terminal KEN box, as clearly seen in the sub-functionalization of BubR1 and Bub1. BubR1 and Bub1 are the result of the duplication of a single multifunctional ancestral protein (Suijkerbuijk et al., 2012). The single Bub1-like proteins in simple chordates, e.g., *Ciona intestinalis*, reveal the likely architecture of the common ancestor of human Bub1 and BubR1 (Figure 4A). After the duplication of the ancestral Bub1-like protein, BubR1 and Bub1 diverged and took on specific roles. This happened on multiple occasions, and tracking the gain and loss of functional modules shows that the evolutionary path of sub-functionalization is remarkably predictable (Figures 4B and 4C). In nearly all cases, the BubR1-like proteins lost their MAD1 binding region and kinase domain (Figure 4D; Figure S3), whereas the Bub1-like proteins lost their N-terminal KEN box and ABBA-KEN-ABBA cassette (Figure 4E; Figure S4).

We noted that conservation of the cassette is always as a complete module in which the inter-motif distance is constrained despite the large evolutionary distances and high rates of mutation/insertion/deletion in the inter-motif regions (Figure 1A). This indicated that the spacing between the motifs might be important to interact with the correct surfaces on APC/C-Cdc20. If the ABBA^272^ motif and the KEN2 box bind to the APC/C-bound Cdc20 (for clarity we refer to this as Cdc20^APC/C^), then modeling this onto the structure of Cdc20 predicts that reducing the spacing would preclude both motifs binding to their respective receptors. (The ABBA motif receptor is between blades 2 and 3 of the WD40 domain, and the KEN box binds the top surface of Cdc20.) To test these predictions, we deleted 12 amino acids between ABBA^272^ and the KEN2 box (Δ280-292) and 17 amino acids between the KEN2 box and ABBA^340^ (Δ319-336); siRNA and rescue experiments showed that neither mutant could function in the SAC (Figure 4F). To determine whether there were any important motifs in these linker regions, we restored the distance between both motifs with a Gly-Gly-Thr linker and found that this restored the checkpoint (Figure 4G), indicating that simply the correct spacing between the ABBA-KEN-ABBA motifs was important for SAC activity.

This led us to conceive a model in which the two ABBA motifs in the cassette stabilize the MCC onto the APC/C by binding the two Cdc20 molecules in the complex. In our model, the ABBA^272^ motif would bind to Cdc20^APC/C^ and ABBA^340^ to Cdc20^MCC^ in the core MCC; therefore, ABBA^272^ would be essential for the MCC to bind the APC/C, but mutations in ABBA^340^ might be compensated by stabilizing the MCC. To test this, we stabilized the binding between Cdc20 and Mad2 in the MCC by co-expressing GBP (GFP binding protein)-Cdc20 and Venus-Mad2 in stable cell lines expressing either WT or ABBA mutant BubR1 and depleting endogenous BubR1 by siRNA (Izawa and Pines, 2015). In this system, stabilizing the MCC promotes mitotic arrest even in the absence of microtubule poisons (Izawa and Pines, 2015). Consistent with our prediction, we found that the stabilized MCC still required ABBA^272^ to arrest cells in mitosis and bind the APC/C, but that ABBA^340^ was no longer essential (Figures 4H–4J).

## Discussion

Here we have identified a conserved cassette in the N-terminal half of BubR1 that plays an essential role in the spindle assembly checkpoint. It contains two ABBA motifs flanking the previously characterized KEN2 box (Burton and Solomon, 2007, Elowe et al., 2010, King et al., 2007, Lara-Gonzalez et al., 2011). Both ABBA motifs bind Cdc20 and, although they are not required for the formation of the MCC, they are essential for the MCC to bind and inhibit active APC/C-Cdc20. Our data are consistent with a model in which ABBA^272^ binds to Cdc20^APC/C^ and ABBA^340^ binds to Cdc20^MCC^.

The ABBA-KEN-ABBA cassette is conserved in all kingdoms and this taxonomic range (eukaryota) is much larger than the previously characterized ABBA motif at position 528 in BubR1 (metazoa) that is also found in Cyclin A (metazoa), Bub1 (metazoa), Clb5 (saccharomycetales), and Acm1 (saccharomycetales). This indicates that the ABBA^272^ and ABBA^340^ motifs may be the original binding partners for the ABBA binding pocket on Cdc20. We note that *C. elegans* appears to be an exception to the conservation of the ABBA-KEN-ABBA cassette, and it will be interesting to determine whether the MCC binds stably to the APC/C through other motifs or whether the dynamics of MCC generation in worms makes stable binding of the MCC to the APC/C less crucial.

On at least five separate occasions during evolution, the N-terminal KEN1 box and the ABBA-KEN-ABBA cassette have been selected together in the BubR1-like proteins, whereas the MAD1 binding regions and kinase domains have been lost, and vice versa in the Bub1-like proteins. This indicates that the ABBA-KEN-ABBA cassette is functionally linked to the N-terminal pseudo-substrate KEN1 box motif. We hypothesize that evolutionary pressures drove the separation of the functionality of the ancestral protein into its substrate modification (kinase domain) plus MAD1 recruitment components, and its MCC inhibitory components (N-terminal KEN1 and the ABBA-KEN-ABBA cassette) to resolve adaptive conflicts between the modules in the ancestral protein (Hittinger and Carroll, 2007).

ABBA^272^ and ABBA^340^ specifically bind Cdc20 but with much lower affinity than the previously described ABBA^528^ (Di Fiore et al., 2015, Kaisari et al., 2016). Binding the MCC to the APC/C through these modular low-affinity binding sites would enable rapid association and dissociation kinetics, thereby allowing MCC binding to the APC/C to respond quickly to checkpoint activity: rapid MCC dissociation would activate the APC/C once the SAC was turned off, but newly produced MCC could quickly inactivate the APC/C should a kinetochore detach before anaphase.

We showed that MCC binds to a second molecule of Cdc20 as a pseudo-substrate inhibitor via the BubR1 D-box and KEN2 box (Izawa and Pines, 2015), but for this to be efficient, the MCC should prefer to bind to APC/C-associated Cdc20 over free Cdc20. Mutating the ABBA^272^ motif abolished binding to the APC/C and inactivated the SAC even though the MCC should still be able to bind Cdc20 as pseudo-substrate inhibitor (since the D-box and KEN2 box on BubR1 are still intact); thus, stable binding of the MCC onto the APC/C-Cdc20 is essential to inhibit the APC/C and arrest cells in mitosis. Mutating the ABBA^340^ motif reduces, but does not abolish, APC/C binding. We propose that the more severe phenotype of the ABBA^272^ mutant is likely to be because it synergizes with the D-box and KEN2 motifs to lock the MCC onto Cdc20^APC/C^, and the ABBA^340^ motif further stabilizes the interaction between the MCC and the APC/C through binding between blades 2 and 3 of Cdc20^MCC^. This would account for the conservation of the longer spacing between the KEN box and ABBA^340^ compared to ABBA^272^.

Two structures of the MCC-APC/C complex were published while this study was under review (Alfieri et al., 2016, Yamaguchi et al., 2016). Both structures provide support for our proposal that the ABBA-KEN-ABBA cassette binds the MCC to the APC/C through binding the two Cdc20 molecules. In both structures, the ABBA^272^ and KEN2 motifs can be visualized binding to Cdc20^APC/C^, but the peptide chain after this is not resolved. The authors of both studies suggested that ABBA^528^ in the C terminus of BubR1 might fold back to bind to Cdc20^MCC^, but we previously showed that mutating ABBA^528^ has only a marginal effect on the SAC (Di Fiore et al., 2015). Instead, our data point to a much simpler solution: if ABBA^272^ binds to Cdc20^APC/C^ and ABBA^340^ binds Cdc20^MCC^, then the ABBA-KEN-ABBA cassette would lie across the MCC-APC/C structure as an almost linear chain (see Figure 2 in Alfieri et al., 2016).

The structures of the MCC-APC/C complex show that Cdc20^APC/C^ is tilted and rotated away from the position where it aligns with APC10 to form the bi-partite D-box receptor (Chao et al., 2012, da Fonseca et al., 2011, Herzog et al., 2009) and that BubR1 has additional contacts with APC2 that would inhibit the binding of an E2 (Alfieri et al., 2016, Yamaguchi et al., 2016). Thus, the MCC inhibits the APC/C in multiple ways, not just as a pseudo-substrate, which agrees with our finding that the ABBA^272^ mutant phenotype is more severe than either the D-box or KEN2 box mutant.

Lastly, our identification of the ABBA motif-Cdc20 interaction as essential to the SAC unveils a potential new way to target the SAC specifically by using small molecules as an alternative to the protein kinase inhibitors currently being developed for clinical trials (Wengner et al., 2016).

## Experimental Procedures

### Cell Culture and Synchronization

HeLa FRT/TO and RPE1-hTERT FRT/TO were transfected using the Flp-In system (Invitrogen) to generate stable cell lines. Cells were grown and synchronized as previously described (Di Fiore et al., 2015). Cells were treated with tetracycline (1 μg/mL, Calbiochem) 12 hr before harvesting. For prometaphase arrest, nocodazole (0.33 μM, Sigma) was added during release from a thymidine block, and 12 hr later, MG132 (10 μM, Calbiochem) was added and cells collected by mitotic shake-off after 2 hr. To obtain metaphase cells, we released cells arrested in prometaphase by dimethyl anastron (DMA) (10 μM, Calbiochem) into reversine (0.5 μM, Cayman Chemical) and MG132 for 2 hr.

### Peptide Pull-Down and Competition

Biotin-conjugated peptides (Selleck Chemicals) were synthesized with the following sequences: wild-type 272 (QMQNNSRITVFDENADEAST), wild-type 340 (VPAVLPSFTPYVEETAQQPV), and wild-type 528 (SKGPSVPFSIFDEFLLSEKK). In the mutant peptides, the third, fourth, and sixth amino acids of the motif were mutated to alanine. Cells arrested in metaphase were lysed in PBS plus 0.5% Triton X-100 and protease inhibitor cocktail (Roche), and the extracts were cleared by centrifugation and were incubated with the biotinylated peptides coupled to streptavidin Ultralink beads (Pierce) for 1 hr at 4°C. For binding to FLAG-Cdc20 mutants, BSA 5 mg/mL was preincubated with the beads and added during the pull-down. After extensive washes, associated proteins were eluted at 65°C for 5 min, separated by SDS-PAGE, and analyzed by immunoblotting on a LI-COR Odyssey scanner.

### Imaging and Analysis

For time-lapse microscopy, cells were seeded and transfected on an 8-well chamber slide (μslide, Ibidi) and the medium replaced with Leibovitz’s L-15 medium (Invitrogen) just before filming. Differential interference contrast (DIC) and fluorescence images were captured every 5 min with a Nikon Eclipse Ti microscope equipped with a Hamamatsu Flash 4.0 sCMOS camera using micromanager software. DIC microscopy was used to monitor mitotic phases. To measure the intensity of kinetochore, we filmed localization cells on a spinning disk microscope (Intelligent Imaging Innovations). Maximum projections of single z sections at NEBD were obtained and the kinetochore intensity quantified using ImageJ.

### Immunoprecipitation

Cells were lysed in 50 mM Tris-HCl (pH 7.8), 150 mM NaCl, 0.2% NP40, and 1 mM EDTA plus protease inhibitor cocktail (Roche) and microcystin (10 nM, LGC Promotech) for 20 min on ice and clarified at 12,000 × *g* for 15 min at 4°C. Complexes were immunoprecipitated for 1 hr at 4°C with anti-FLAG (M2, Sigma) covalently coupled to Dynabeads protein G (Invitrogen). After five washes in lysis buffer, proteins were eluted from beads by incubating 5 min at 65°C in SDS-PAGE sample buffer.

### rMCC Purification and In Vitro Ubiquitylation Assays

MultiBac constructs encoding the sequences for Cdc20, SBP-BubR1 (WT or mutants), His_6_-Mad2, and Bub3 were used to infect Sf9 insect cells. Recombinant MCC was purified by biotin elution from Strep Agarose Beads (Invitrogen) followed by Ni-NTA beads (QIAGEN). Purified rMCC was analyzed by silver staining (Sigma Aldrich) after SDS-PAGE. SBP-Cdc20 was expressed and purified from Sf9 cells. In vitro ubiquitylation assays were performed as described previously (Izawa and Pines, 2015), except that an APC4 monoclonal antibody was used to isolate the apo-APC/C. APC/C immunoprecipitated from Cdc20-depleted mitotic HeLa cell extract was pre-incubated on ice with rMCC and recombinant SBP-Cdc20. Securin labeled with fluorescent maleimide dyes (LI-COR) was used as a substrate. Reactions were started by the addition of E1 and E2 enzymes, ubiquitin and ATP, and incubated at 37°C for 15 min. Reaction products were separated by SDS-PAGE and visualized using a LI-COR Odyssey scanner. Levels of Cdc20 and other components were quantified by immunoblotting.

### Sequence and Evolutionary Analysis

Bub1-like proteins were retrieved from 13 eukaryotic organisms: *H. sapiens*, *C. intestinalis*, *D. melanogaster*, *A. mellifera*, *C. elegans*, *M. brevicollis*, *U. maydis*, *S. pombe*, *A. gossypii*, *S. cerevisiae*, *P. infestans*, *A. thaliana*, and *M. pusilla*. Species were chosen based on the Bub1-like protein family tree constructed by Suijkerbuijk et al. (2012) to create subclades of two species: one containing a single copy Bub1-like protein and one containing two or more Bub1-like proteins. Proteins were split into three groups: (1) single copy Bub1-like proteins, (2) duplicated Bub1-like proteins, and (3) duplicated BubR1-like proteins. The retained modules, inconclusively retained modules, and potentially absent modules in Bub1-like proteins were defined as described in Supplemental Experimental Procedures. Absent motifs could not be detected by the search criteria but may still be present; for example, the structure of the D-box binding pocket of *S. pombe* Cdc20 is occupied by a non-consensus peptide from Mad3 (Chao et al., 2012).

## Author Contributions

B.D.F., C.W., and J.P. designed the experiments. B.D.F. and C.W. performed the experiments. N.E.D. performed the sequence and evolutionary analysis. All authors contributed to the analysis of the results. B.D.F., N.E.D., and J.P. wrote the paper.

## Supplemental Information

Document S1. Supplemental Experimental Procedures and Figures S1–S3

Table S1, Related to Figure 4. Search Criteria for Domains Shown in Figure 4 Are Detailed

Document S2. Article plus Supplemental Information
