# Supplementary material for: The Mitotic Checkpoint Complex Requires an Evolutionary Conserved Cassette to Bind and Inhibit Active APC/C
Source: Mol Cell. 2016 Dec 15;64(6):1144–53. doi: 10.1016/j.molcel.2016.11.006 (PMC5179498; doi:10.1016/j.molcel.2016.11.006)
Supplement: Document S1. Supplemental Experimental Procedures and Figures S1–S3 [file mmc1.pdf]

Molecular Cell, Volume *64*

Supplemental Information

The Mitotic Checkpoint Complex Requires an Evolutionary Conserved Cassette to Bind and Inhibit Active APC/C

Barbara Di Fiore, Claudia Wurzenberger, Norman E. Davey, and Jonathon Pines

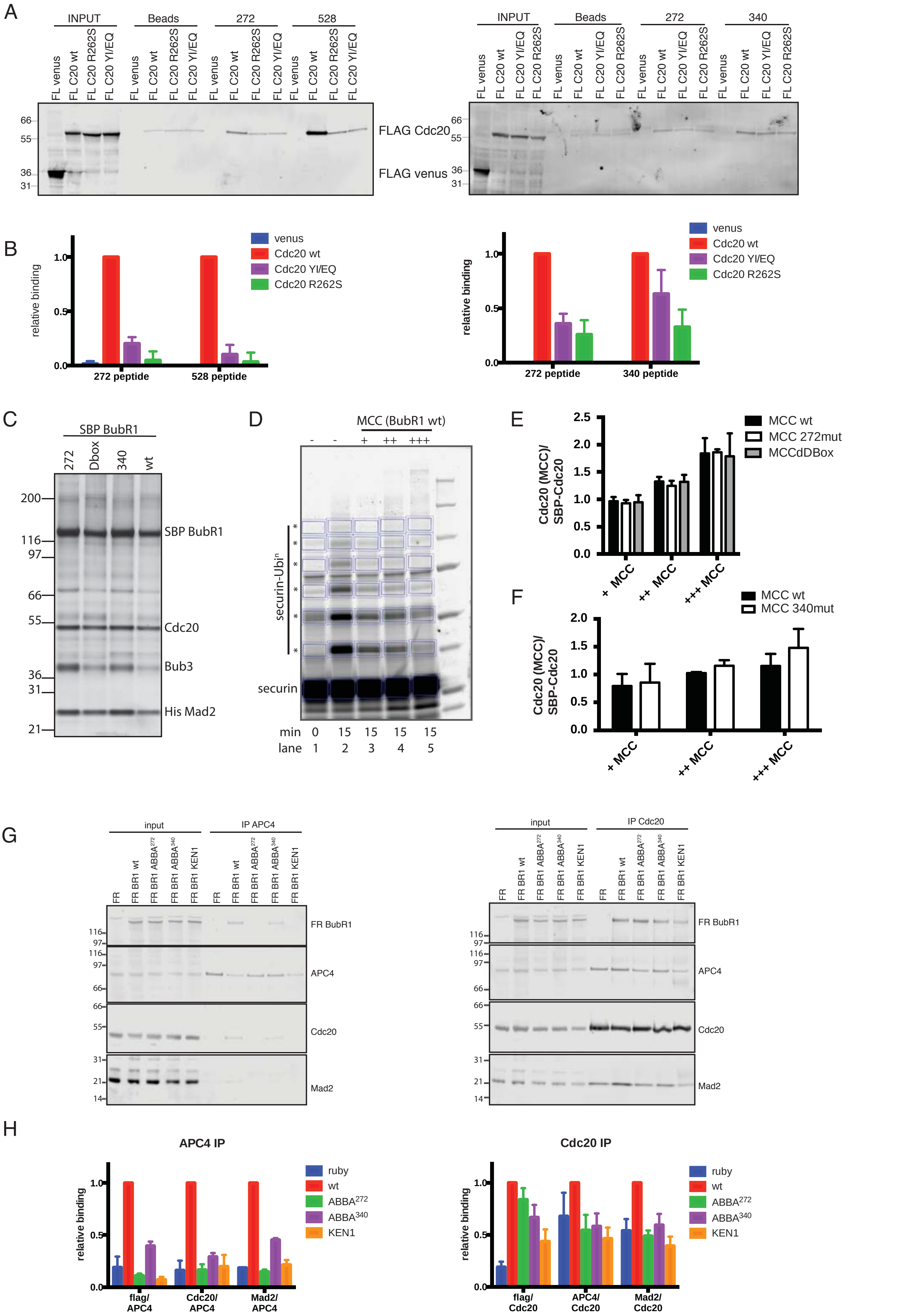

A
INPUT
Beads
272
528
340
FL venus
FL C20 wt
FL C20 R262S
FL C20 YI/EQ
FLAG Cdc20
FLAG venus
B
relative binding
venus
Cdc20 wt
Cdc20 YI/EQ
Cdc20 R262S
272 peptide
528 peptide
340 peptide
C
SBP BubR1
272
Dbox
340
wt
Cdc20
Bub3
His Mad2
D
MCC (BubR1 wt)
securin-Ubi^{n}
securin
min
lane
E
Cdc20 (MCC)/ SBP-Cdc20
MCC wt
MCC 272mut
MCCdDBox
+ MCC
++ MCC
+++ MCC
F
MCC wt
MCC 340mut
G
input
IP APC4
IP Cdc20
FR
FR BR1 wt
FR BR1 ABBA^{272}
FR BR1 ABBA^{340}
FR BR1 KEN1
FR BubR1
APC4
Mad2
H
APC4 IP
Cdc20 IP
ruby
wt
ABBA^{272}
ABBA^{340}
KEN1
flag/ APC4
Cdc20/ APC4
Mad2/ APC4
flag/ Cdc20
APC4/ Cdc20
Mad2/ Cdc20

Figure S1

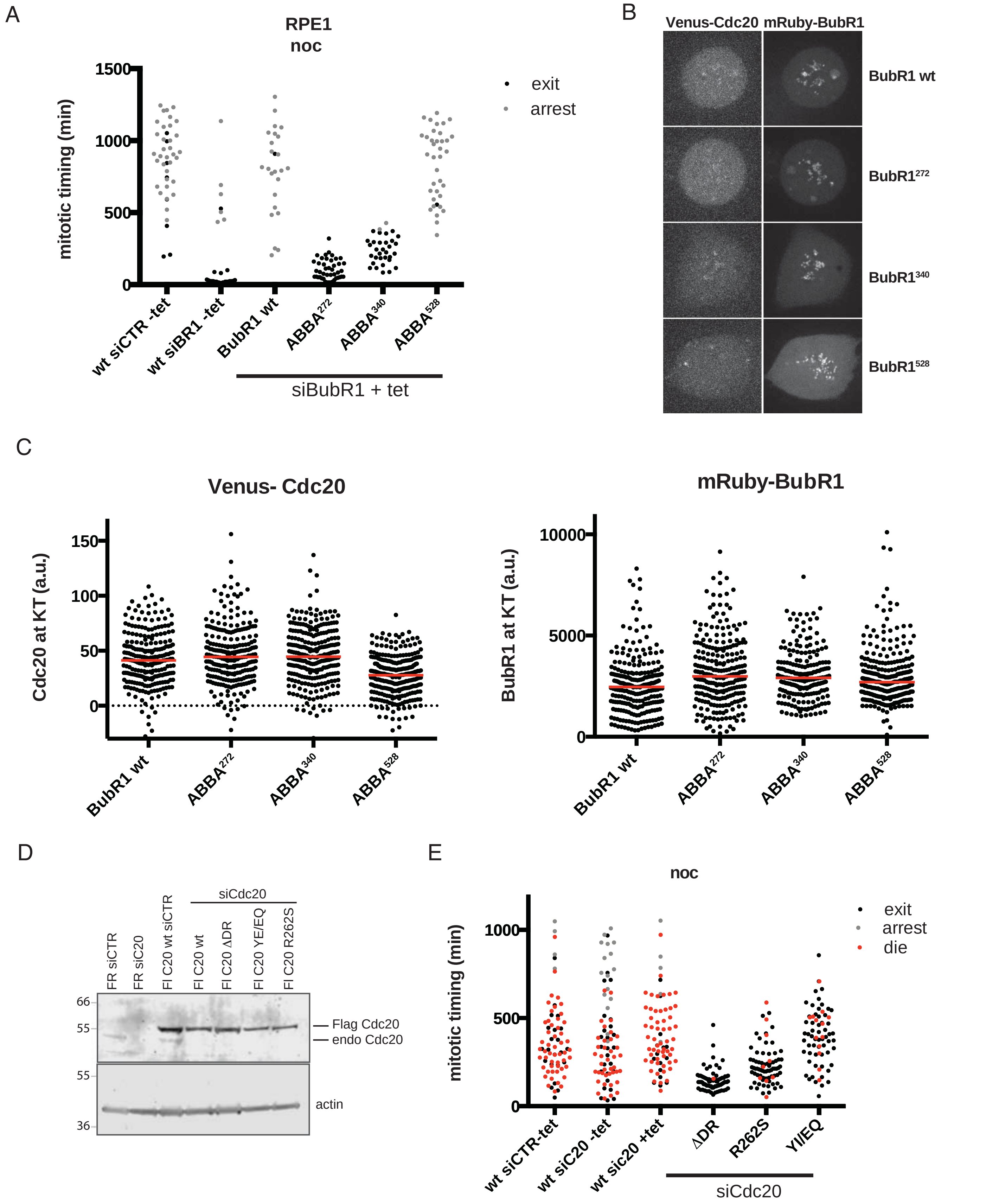
A
RPE1
noc
mitotic timing (min)
1500
1000
500
0
exit
arrest
wt siCTR -tet
wt siBR1 -tet
BubR1 wt
ABBA272
ABBA340
ABBA528
siBubR1 + tet
B
Venus-Cdc20
mRuby-BubR1
BubR1 wt
BubR1272
BubR1340
BubR1528
C
Venus- Cdc20
Cdc20 at KT (a.u.)
150
100
50
0
BubR1 wt
ABBA272
ABBA340
ABBA528
mRuby-BubR1
BubR1 at KT (a.u.)
10000
5000
0
BubR1 wt
ABBA272
ABBA340
ABBA528
D
siCdc20
FR siCTR
FR siC20
Fl C20 wt siCTR
Fl C20 wt
Fl C20 ΔDR
Fl C20 YE/EQ
Fl C20 R262S
66
55
55
36
Flag Cdc20
endo Cdc20
actin
E
noc
mitotic timing (min)
1000
500
0
exit
arrest
die
wt siCTR-tet
wt siC20 -tet
wt sic20 +tet
ΔDR
R262S
YI/EQ
siCdc20

Figure S2

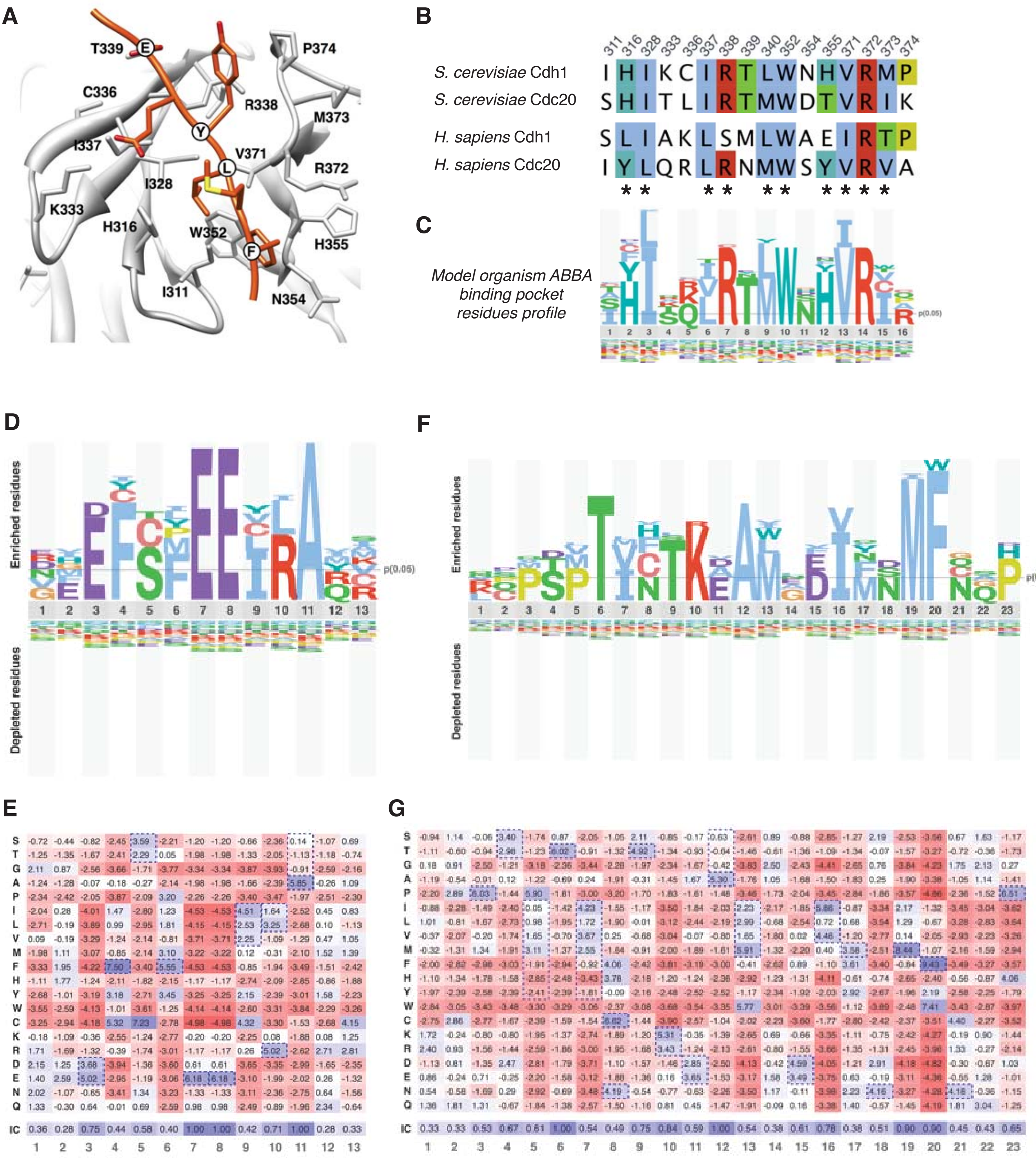
A
T339
P374
C336
R338
M373
I337
V371
R372
I328
K333
H316
W352
H355
I311
N354
B
S. cerevisiae Cdh1
S. cerevisiae Cdc20
H. sapiens Cdh1
H. sapiens Cdc20
311 316 328 333 336 337 338 339 340 352 354 355 371 372 373 374
I H I K C I R T L W N H V R M P
S H I T L I R T M W D T V R I K
S L I A K L S M L W A E I R T P
I Y L Q R L R N M W S Y V R V A
C
Model organism ABBA binding pocket residues profile
D
Enriched residues
Depleted residues
p(0.05)
F
Enriched residues
Depleted residues
p(0.05)
E
G

Fig S3

Supplemental Figure legends

Supplemental Figure 1 - Related to Figure 1 and 2

(A) Peptide pull-downs were performed as in Fig1C, with extracts from HeLa FRT/TO cell lines expressing siRNA-resistant tetracyclin-inducible wild-type (wt) or mutant FLAG-tagged Cdc20 (FL C20). Endogenous Cdc20 was depleted by siRNA. (B) Quantification of the peptide pull-downs shown in panel A. Mean and SEM from 3 independent experiments. The reduction in binding of the mutant $ABBA^{340}$ to Cdc20 is more variable than mutant $ABBA^{272}$, potentially due to the presence of a non-canonical Pro at position 3 of $ABBA^{340}$, which might therefore have evolved other residues to stabilise the binding to Cdc20. (C) Silver stain of recombinant MCC containing wild type or mutant SBP-BubR1 purified from baculovirus-infected insect cells and analysed by SDS PAGE. (D) Ubiquitylation assay similar to Fig 2A is shown with the bands quantified for Fig 2B highlighted in blue. To calculate relative ubiquitylation, quantification was performed as follows: the fluorescence value of the bands in lane 1 were subtracted from each band of lane 2 to 5 migrating with the same mobility, and the sum of the 6 bands (marked with asterisks) designated as ubiquitylated securin. Relative ubiquitylation was calculated by dividing the values of lanes 3 to 5 by the value obtained in lane 2, considered to be the maximum ubiquitylation, in the absence of MCC. (E-F) Quantification of Cdc20 levels in the ubiquitylation reactions in Fig 2A as analysed by quantitative immunoblotting. (G) HeLa FRT/TO cell lines stably expressing inducible siRNA-resistant FLAG-mRuby (FR)-BubR1, wt or mutants, were transfected with siRNA to knockdown endogenous BubR1. Anti-APC4 (left) and anti-Cdc20 (right) immunoprecipitations from nocodazole-arrested cells were analysed by immunoblotting and visualised on a Li-COR Odyssey scanner. (H) Mean and SEM of protein levels from 4 independent experiments shown in panel G. Note that although the KEN1 BubR1 mutant is unable to form the MCC it can still bind Cdc20 through its $ABBA^{528}$ motif.

Supplemental Figure 2 - Related to Figure 3

(A) RPE1 FRT/TO cell lines stably expressing inducible siRNA-resistant FLAG-mRuby tagged BubR1, wild type or mutants, were transfected with siRNA against BubR1 or control siRNA (siCTR) and the mitotic timing measured in cells treated with nocodazole (0.33μM). At least 30 cells per condition were analysed. Results representative of 3 independent experiments. (B) Representative images of RPE1 FRT/TO cell lines expressing Cdc20 endogenously-tagged at one allele with the Venus fluorescent protein, and stably expressing siBubR1-resistant inducible FLAG-mRuby BubR1, wt or mutant, after depleting endogenous BubR1 with siRNA and treated with nocodazole (0.33μM). Images were acquired at the spinning disk with 10% laser power to reduce photo damage/fluorescence bleaching. (C) Kinetochore levels of Cdc20 were measured in cells shown in Fig S2B at the first time point after Nuclear Envelope Breakdown, selecting the kinetochores based on mRuby-BubR1 signal (right panel) and measuring the same area for Venus-Cdc20 images (left panel). At least 250 kinetochores from 15 cells were quantified for each condition. (D) HeLa FRT/TO cell lines stably expressing inducible siRNA-resistant FLAG-tagged Cdc20, wild type, D-box receptor (ΔDR) or ABBA receptor (R262S and YI/EQ) mutants, were transfected with siRNA against Cdc20 or control siRNA

(siCTR). FLAG mRuby (FR) cell lines are used as control. Immunoblotting analysis shows the expression levels of ectopic Cdc20 compared with endogenous Cdc20, and the efficiency of depletion. Actin is a loading control. (E) HeLa FRT/TO cells characterised in panel D were treated with nocodazole (0.33μM) and the mitotic timing measured. At least 70 cells per condition were analysed. Representative of 3 independent experiments. Please note that although cells expressing the YI/EQ mutant do not exit mitosis as fast as R262S, they still slip through the nocodazole arrest; by contrast most cells expressing wild type Cdc20 die during the arrest.

Supplemental Figure 3 - Related to Figure 4

(A) Structure of the ABBA motif from yeast Acm1 bound to the ABBA motif binding pocket in yeast Cdh1 labelled with the positions of the residues (Burton et al., 2011; He et al., 2013). (B) ABBA motif binding pocket residues of the yeast and human Cdc20-like proteins removed from a Clustal Omega alignment of the 32 CDC20-like proteins in twelve model eukaryotic organisms: Arabidopsis thaliana, Caenorhabditis elegans, Chlamydomonas reinhardtii, Dictyostelium discoideum, Drosophila melanogaster, Homo sapiens, Monosiga brevicollis, Phytophthora infestans (strain T30-4), Plasmodium falciparum (isolate 3D7), Saccharomyces cerevisiae (strain ATCC 204508 / S288c), Schizosaccharomyces pombe (strain 972 / ATCC 24843) and Xenopus tropicalis. (C) Relative binomial logo of the residues in the ABBA motif binding pocket from the 32 CDC20-like proteins. (D) Relative binomial logo created from the peptides within the Bub1/BubR1 alignment (see http://slim.ucd.ie/abbakenabba/ for alignments) aligned to the experimentally characterised instances of the GLEB motif core (the motif has extensions but they are not at a constant spacing) (Larsen et al., 2007). Aligned GLEBS motif peptides in the Bub1/BubR1 alignment: VGEFSFEEIRAEV, VGEFQPEEILAAC, RLEFSLEEVLAIS, RDDACYEERRAVR, QFELTLEELIARR, NMDQSMEEVRAQV, NIEFSPEEYRAYS,GVEYSPEEILARK, GEERSMEECRAQR, GEAISFEESRARA, ESEFSFEELRAQK, EEDCTFEELRANM, DHEFCIEEILALA, DEEFNTEEILAMI. (E) PSI-BLAST PSSM (position specific scoring matrix) of the core GLEBS motif peptides (as defined in panel D). PSSM created using the PSI-BLAST PSSM creation protocol including sequence weighting and pseudocounts (Altschul and Koonin, 1998; Schäffer et al., 2001). PSSM significance cut-off with p-value of 0.00001 calculated based on peptide randomisation. Bub1/BubR1 proteins were scanned with PSSM and a GLEBS motif is defined as present if a peptide scores above the PSSM significance cut-off. (F) Relative binomial logo created from the peptides within the Bub1/BubR1 alignment (See http://slim.ucd.ie/abbakenabba/_for alignments) aligned to the experimentally characterised Mad1 binding region (London and Biggins, 2014). Aligned Mad1 binding region peptides in the Bub1/BubR1 alignment: VQPSPTVHTKEALGFIMNMFQAP, RSPTVTMFSRDAMKEVYSMFNQH, RSPTVTAFSKDAINEVFSMFNQH, QDPTMTICTKEAWGDIMSMFSGG, NCPSPTINTKAAMADIMAMFGAP, LRMSPTLVTKAAVLEVENMFNGD, LGQSLTVNTKEAMSVVQDMWRSP, KPSSPTVCTKEAMGEIFGMFQKP, KPPSPTIHTKAALADILDIFNQP, GLVDPTVNLKEAMEDINNMFGEP, EQEDMTINTRVALEDINNMFCSP. (G) PSI-BLAST PSSM (position specific scoring matrix) of the Mad1 binding region peptides (as defined in panel F). PSSM

created as described in panel D. PSSM significance cut-off with p-value of 0.00001 calculated based on peptide randomisation. Bub1/BubR1 proteins were scanned with PSSM and a Mad1 binding region is defined as present if a peptide scores above the PSSM significance cut-off.

Supplemental Table 1 – Related to Figure 4

Search criteria for domains shown in Figure 4 are detailed.

Supplemental Experimental Procedures

Plasmid generation

BubR1, Cdc20 and their mutants were cloned into a modified version of pcDNA5/FRT/TO (Invitrogen), which contained at the N-terminus either FLAG tag alone or linked to the mRuby or Cerulean fluorescent proteins. Mutants and siRNA-resistant constructs were generated using overlapping PCR. For the $ABBA^{272}$ mutant the third, fourth and sixth positions were replaced with alanine, and for the $ABBA^{340}$ motif mutant the first, third, fourth and sixth positions were replaced with alanine. To test the role of the spacing between the motifs in the cassette, residues 280-292 and 319-336 were deleted. In the rescue plasmids a repeated Gly-Gly-Thr linker was introduced to restore the distance but not the sequence between motifs. All plasmids were verified by sequencing. Details of cloning are available on request.

RNA interference

siRNA duplex against BubR1 (GAUGGUGAAUUGUGGAAUA, Dharmacon), Cdc20 (CGGAAGACCUGCCGUUACAUU, Dharmacon) and ON-TARGETplus non-targeting siRNA pool (Sigma) were transfected at a final concentration of 50nM with RNAimax (Invitrogen) according to the manufacturer's protocol. For immunoprecipitation experiments a single siRNA transfection was performed before a single thymidine block protocol. For siRNA rescue experiments, a double siRNA transfection/double thymidine synchronization protocol was followed, with the first siRNA transfection performed before the first thymidine addition and the second siRNA transfection about 6 hours after release from the first thymidine block.

Immunoblotting

After electrophoresis on 4-12% gradient Bis-Tris acrylamide gels, proteins were transferred to a PVDF membrane. Membrane saturation and all the following incubation steps were carried out in 5% low fat milk in PBS-Tween 0.1%. The following primary antibodies were used at the indicated dilutions: FLAG (M2, Sigma, 1:1000), Mad2 (Bethyl Laboratories, 1:1000), actin (AC-15, Sigma, 1:1000), Cdc20 (Bethyl Laboratories, 1:500), APC4 (monoclonal antibody raised against a C-terminal peptide, 1:500), BubR1 (Bethyl Laboratories1:500), Bub3 (Bethyl Laboratories, 1:500), Bub1 (Abcam, 1:3000). IRDye680 and IRDye800CW (LI-COR)-conjugated secondary antibodies were used at 1:10000. The antibody signal was detected using the Odyssey infrared imaging system (LI-COR) for quantitative

immunoblotting.

Relative binomial logo construction

Relative binomial logos were calculated as $prob^{aa} = binomial(k,n,p)$ where k is the observed residue count at each position for a residue, n is the number of the instances of motifs and p is the background frequency of the residue in the intrinsically disordered regions of the human proteome. The grey line annotated as p(0.05) signifies the height of a amino acid that has a $prob^{aa}$ of 0.05. The data are split based on whether a residue is depleted or enriched at a particular position. A positive score (above the central partition containing the residue offset numbering) denotes that a residues is enriched, a negative score (below the partition) denotes that a residue is depleted, and score of zero suggests that a residue is occurring at the expected frequency.

Affinity and specificity determinants of the ABBA motif

List of experimentally characterised ABBA motif instances

Gene	Protein	Sequence	Reference
H. sapiens CDC20			
Bub1	Mitotic checkpoint kinase BUB1	slssa 527 **FHVFED** 532 gnken	(Di Fiore et al., 2015)
BubR1	Mitotic checkpoint kinase BUB1 beta	gpsvp 528 **FSIFDE** 533 fllse	(Di Fiore et al., 2015; Díaz-Martínez et al., 2015)
BubR1	Mitotic checkpoint kinase BUB1 beta	qnnsr 272 **ITVFDE** 277 nadea	*This study*
BubR1	Mitotic checkpoint kinase BUB1 beta	avlps 340 **FTPYVE** 345 etarq	*This study*
CCNA2	Cyclin-A2	skqpa 99 **FTIHVD** 104 eaeke	(Di Fiore et al., 2015)
S. cerevisiae Cdh1			
Acm1	APC/C-CDH1 modulator 1	skaaq 61 **FMLYEE** 66 taeer	(He et al., 2013) *{Burton:2011dl}*
S. cerevisiae Cdc20			
Clb5	S-phase entry cyclin-5	vsavq 99 **KRQIYND** 105 rtaae	(Lu et al., 2014)
Mad3	SAC component MAD3	dvnlt 258 **KNNVFVD** 264 geesd	*By homology* [a]
Mad3	SAC component MAD3	rpyde 330 **KLPIFRD** 336 sigrs	*By homology* [a]

[a] Mapped by homology from the human BubR1 ABBA motifs described in this study. The loss of the position +1 hydrophobic specificity and affinity determinant and the gain of a position -1 basic determinant observed and explained by Lu *et al* 2014 for Clb5 is once again observed in both Mad3 ABBA motifs.

Structural, sequence and evolutionary analysis of the available ABBA motif instances indicate that the metazoan Cdc20 and fungal Cdh1 ABBA motif binding pockets recognise peptides containing the consensus ϕ-x-ϕ-ϕ-x-D/E. In metazoa, the P1 position has been observed to allow a hydrophobic amino acid from the set of I/L/V/M/F. P3 position prefers an I/L/V, however the second human ABBA of the ABBA-KEN-ABBA cassette has a conservative substitution of another aliphatic residue – proline - in this position. The P4 position has a more constrained preference showing a strong enrichment for an aromatic residue, only a handful of instances diverge from this. As previously observed and explained by Lu *et al.,* 2014 for the Clb5 ABBA motif, the fungal Cdc20 ABBA motif binding pocket prefers K-x-x-ϕ-ϕ-x-D/E as a result of a change in the ABBA motif binding pocket.

Both ABBA motifs (mapped by homology) of the ABBA-KEN-ABBA cassette in yeast Mad3 match this preference.

The four original ABBA motifs in Cyclin A, Bub1, BubR1 and Acm1 indicated a consensus of Fx[ILV][FHY]x[DE]. This consensus is strongly conserved in all four instances but not in the ABBA-KEN-ABBA cassette where other hydrophobic residues are allowed, particularly in the P1 positions. The determinants may be less strictly required due to the multivalency of the interaction permitting a sub-optimal motif and may explain why the ABBA motifs of the ABBA-KEN-ABBA cassette are weaker binders than the original ABBA motifs: both contain an infrequently observed and potentially weaker amino acid at a single position: P1 for ITVFDE and P3 for FTPYVE.

Key residues in the potential ABBA binding pocket of human Cdh1, conserved across all metazoan Cdc20s and fungal Cdh1, strongly differ from the consensus – P338 and P355 (Figure S3A-B - yeast Cdh1 numbering). The replacement of the Arginine at P338 with a small amino acid, serine, would have a large effect on the binding pocket. Conversely, the ABBA binding pocket in yeast Cdh1 has been largely conserved relative to the human Cdc20 pocket, and the yeast Cdc20 binding pocket has diverged only in the ABBA motif P1 contacting positions. This would suggest that the metazoan Cdh1 ABBA binding pocket has had a large shift in specificity and no longer recognises "canonical" ABBA motifs, or, it no longer exists as a binding site for any motif. In agreement with this the human ABBA motifs BUB1 527-532, BUB1B 528-533 and Cyclin A 99-104 do not bind human Cdh (Di Fiore et al., 2015). .

Search criteria for homology mapping of functional modules in the Bub1-like protein family

The search criteria to define the conservation of the functional modules in the Bub1-like proteins used to construct figure 4C in the main text. See Table S1 for details of the homology-mapped modules.

Bub1-like proteins retrieved from 13 eukaryotic organisms were split into 3 groups: (i) single copy Bub1-like proteins; (ii) duplicated Bub1-like proteins; and (iii) duplicated BubR1-like proteins. Proteins from group (ii) and (iii) were aligned to the proteins from group (i) with the MSAProbs multiple sequence alignment tool to create two alignments (See http://slim.ucd.ie/abbakenabba/).

<u>*ABBA motif*</u>

Peptides from the Bub1/BubR1 alignment (See http://slim.ucd.ie/abbakenabba/) aligned to the experimentally characterised instances of the motif and matching the ϕxϕϕx[DE] or Kxxϕϕx[DE] consensus are defined as retained modules. Consensus definition is described above.

<u>*KEN box*</u>

Peptides from the Bub1/BubR1 alignment (See http://slim.ucd.ie/abbakenabba/) aligned to the experimentally characterised KEN box motif instances and matching the KEN consensus are defined as retained modules. Peptides from the alignment aligned to the experimentally characterised instances of the motif and matching the xEN consensus are defined as inconclusively retained modules.

Consensus derived from experimentally validated instances at the APC/C degron repository (http://slim.ucd.ie/apc/).

D box

Peptides from the Bub1/BubR1 alignment (See http://slim.ucd.ie/abbakenabba/) aligned to the experimentally characterised instances of the motif and matching the RxxLxx[ILMVK] consensus are defined as retained modules. Peptides aligned to the experimentally characterised instances of the motif and matching the RxxFxx[ILMVK] consensus are defined as inconclusively retained modules.

Consensus derived from experimentally validated instances at the APC/C degron repository (http://slim.ucd.ie/apc/).

GLEBS

See Fig S3D-E

MAD1 binding region

See Fig S3F-G

TPR domain

Presence in the Bub1/BubR1 protein of an Interpro Mad3/Bub1 homology region 1 (IPR013212) domain

Kinase domain

Presence in the Bub1/BubR1 protein of an Interpro Protein kinase-like domain (IPR011009) domain

Supplemental References

Altschul, S.F., and Koonin, E.V. (1998). Iterated profile searches with PSI-BLAST--a tool for discovery in protein databases. Trends in Biochemical Sciences *23*, 444–447.

Burton, J.L., Xiong, Y., and Solomon, M.J. (2011). Mechanisms of pseudosubstrate inhibition of the anaphase promoting complex by Acm1. Embo J. *30*, 1818–1829.

Di Fiore, B., Davey, N.E., Hagting, A., Izawa, D., Mansfeld, J., Gibson, T.J., and Pines, J. (2015). The ABBA motif binds APC/C activators and is shared by APC/C substrates and regulators. Dev. Cell *32*, 358–372.

Díaz-Martínez, L.A., Tian, W., Li, B., Warrington, R., Jia, L., Brautigam, C.A., Luo, X., and Yu, H. (2015). The Cdc20-binding Phe box of the spindle checkpoint protein BubR1 maintains the mitotic checkpoint complex during mitosis. J. Biol. Chem. *290*, 2431–2443.

He, J., Chao, W.C.H., Zhang, Z., Yang, J., Cronin, N., and Barford, D. (2013). Insights into degron recognition by APC/C coactivators from the structure of an Acm1-Cdh1 complex. Mol. Cell *50*, 649–660.

Larsen, N.A., Al-Bassam, J., Wei, R.R., and Harrison, S.C. (2007). Structural analysis of Bub3 interactions in the mitotic spindle checkpoint. Proceedings of the National Academy of Sciences of the United States of America *104*, 1201–1206.

London, N., and Biggins, S. (2014). Mad1 kinetochore recruitment by Mps1-mediated phosphorylation of Bub1 signals the spindle checkpoint. Genes & Development *28*, 140–152.

Lu, D., Hsiao, J.Y., Davey, N.E., Van Voorhis, V.A., Foster, S.A., Tang, C., and Morgan, D.O. (2014). Multiple mechanisms determine the order of APC/C substrate degradation in mitosis. J. Cell Biol. *207*, 23–39.

Schäffer, A.A., Aravind, L., Madden, T.L., Shavirin, S., Spouge, J.L., Wolf, Y.I., Koonin, E.V., and Altschul, S.F. (2001). Improving the accuracy of PSI-BLAST protein database searches with composition-based statistics and other refinements. Nucleic Acids Research *29*, 2994–3005.
