# Supplementary material for: The Mitotic Checkpoint Complex Requires an Evolutionary Conserved Cassette to Bind and Inhibit Active APC/C
Source: Mol Cell. 2016 Dec 15;64(6):1144–53. doi: 10.1016/j.molcel.2016.11.006 (PMC5179498; doi:10.1016/j.molcel.2016.11.006)
Supplement: Document S2. Article plus Supplemental Information [file mmc3.pdf]

Short Article

Molecular Cell

The Mitotic Checkpoint Complex Requires an Evolutionary Conserved Cassette to Bind and Inhibit Active APC/C

Graphical Abstract

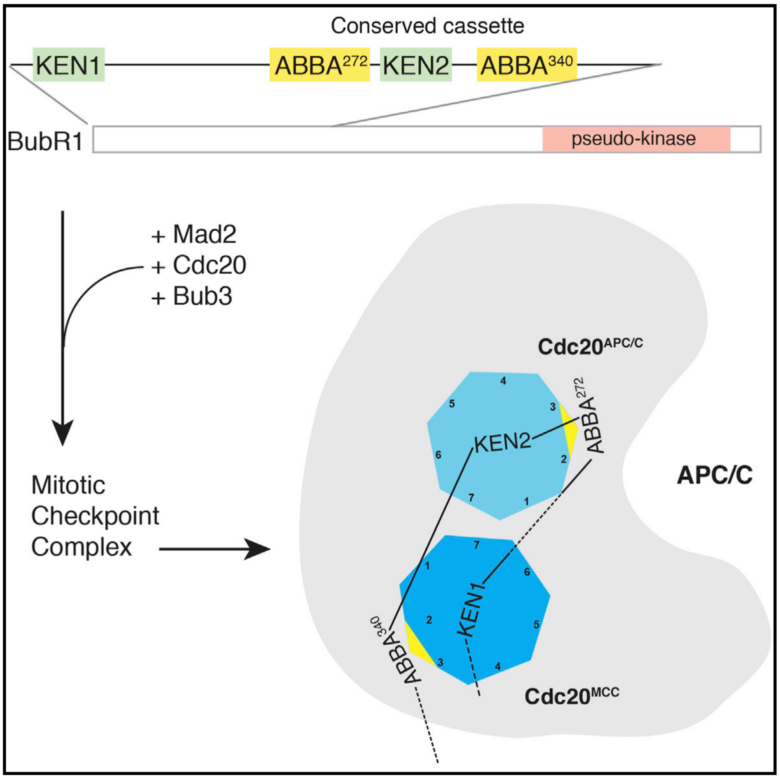

Authors

Barbara Di Fiore,
Claudia Wurzenberger,
Norman E. Davey, Jonathon Pines

Correspondence

jon.pines@icr.ac.uk

In Brief

Di Fiore et al. show that two ABBA motifs in BubR1 are crucial for the Spindle Assembly Checkpoint because they are needed for the Mitotic Checkpoint Complex (MCC) to bind and inhibit the Anaphase Promoting Complex/ Cyclosome (APC/C). Their results show how the MCC inhibits active APC/C.

Highlights

- The N-terminal half of BubR1 contains two ABBA motifs that bind Cdc20
- The motifs are required for the MCC to bind and inhibit active APC/C-Cdc20
- These ABBA motifs are essential for the spindle assembly checkpoint
- The ABBA motifs flank a KEN box to form a cassette highly conserved in evolution

Di Fiore et al., 2016, Molecular Cell *64*, 1144–1153
December 15, 2016 © 2016 The Authors. Published by Elsevier Inc.
http://dx.doi.org/10.1016/j.molcel.2016.11.006

Molecular Cell
Short Article

The Mitotic Checkpoint Complex Requires an Evolutionary Conserved Cassette to Bind and Inhibit Active APC/C

Barbara Di Fiore,[1] Claudia Wurzenberger,[1] Norman E. Davey,[2] and Jonathon Pines[1,3,4,*]
[1]The Gurdon Institute and Department of Zoology, University of Cambridge, Cambridge CB2 1QN, UK
[2]Conway Institute of Biomolecular and Biomedical Sciences, University College Dublin, Dublin 4, Ireland
[3]Division of Cancer Biology, The Institute of Cancer Research, 237 Fulham Road, London SW3 6JB, UK
[4]Lead Contact
*Correspondence: jon.pines@icr.ac.uk
http://dx.doi.org/10.1016/j.molcel.2016.11.006

SUMMARY

The Spindle Assembly Checkpoint (SAC) ensures genomic stability by preventing sister chromatid separation until all chromosomes are attached to the spindle. It catalyzes the production of the Mitotic Checkpoint Complex (MCC), which inhibits Cdc20 to inactivate the Anaphase Promoting Complex/Cyclosome (APC/C). Here we show that two Cdc20-binding motifs in BubR1 of the recently identified ABBA motif class are crucial for the MCC to recognize active APC/C-Cdc20. Mutating these motifs eliminates MCC binding to the APC/C, thereby abolishing the SAC and preventing cells from arresting in response to microtubule poisons. These ABBA motifs flank a KEN box to form a cassette that is highly conserved through evolution, both in the arrangement and spacing of the ABBA-KEN-ABBA motifs, and association with the amino-terminal KEN box required to form the MCC. We propose that the ABBA-KEN-ABBA cassette holds the MCC onto the APC/C by binding the two Cdc20 molecules in the MCC-APC/C complex.

INTRODUCTION

Genomic stability in mitosis is ensured by the Spindle Assembly Checkpoint (SAC), which monitors proper chromosome attachment to the mitotic spindle. The SAC works through unattached kinetochores catalyzing the production of the Mitotic Checkpoint Complex (MCC) (Hardwick et al., 2000; Sudakin et al., 2001), which inhibits Cdc20 to prevent the Anaphase Promoting Complex/Cyclosome (APC/C) ubiquitin ligase from recognizing securin and Cyclin B1 (Fang et al., 1998; Hwang et al., 1998; Kim et al., 1998; Primorac and Musacchio, 2013). According to the accepted "Mad2 Template" model (De Antoni et al., 2005), Mad1-Mad2 heterodimers on unattached kinetochores catalyze a conformational change in a second Mad2 protein that allows it to bind Cdc20 (Luo et al., 2002; Sironi et al., 2002), and this Mad2-Cdc20 complex binds BubR1-Bub3 to form the hetero-tetrameric MCC (Chao et al., 2012; Herzog et al., 2009; Sudakin et al., 2001). Mad2 in the MCC prevents Cdc20 from binding and activating the APC/C (Izawa and Pines, 2012; Zhang and Lees, 2001) and the MCC itself acts as a pseudo-substrate inhibitor (Burton and Solomon, 2007; Chao et al., 2012; Lara-Gonzalez et al., 2011). Cdc20 binding to Mad2 is mutually exclusive with binding to the APC/C (Izawa and Pines, 2012); yet, we and others showed that, even after the APC/C is activated by binding Cdc20, the SAC can rapidly inhibit APC/C-Cdc20 should kinetochore-microtubule attachment be perturbed (Clute and Pines, 1999; Dick and Gerlich, 2013). How the SAC could inhibit the active APC/C-Cdc20 complex was unclear, but it was hypothesized (Burton and Solomon, 2007; Primorac and Musacchio, 2013) and recently shown (Izawa and Pines, 2015) that the MCC can recognize a second Cdc20 molecule through its D-box and KEN box degron receptor sites (Izawa and Pines, 2015).

Although the MCC only binds weakly to Cdc20 (Izawa and Pines, 2015), it binds to APC/C-Cdc20 with sufficient affinity to be co-purified by gel filtration or immunoprecipitation (Herzog et al., 2009; Morrow et al., 2005; Nilsson et al., 2008; Sudakin et al., 2001). How the MCC binds stably to the APC/C is not known: it must involve APC/C-bound Cdc20 because mutating the latter's isoleucine arginine (IR) tail destabilizes interaction with the MCC (Hein and Nilsson, 2014), but mutating the pseudo-substrate D-box and KEN box sites on BubR1 does not prevent MCC binding (Izawa and Pines, 2015). Thus, additional sites of interaction between the MCC and APC/C-Cdc20 must exist.

We and others recently described a conserved motif in the C-terminal half of BubR1 starting at residue 528 that binds to Cdc20 and is required for BubR1 to recruit Cdc20 to kinetochores (Di Fiore et al., 2015; Han et al., 2014; Lischetti et al., 2014). We named this the ABBA motif because it is conserved in Cyclin A, Bub1, BubR1, and Acm1 (Di Fiore et al., 2015), although it has also been called the Phe box in BubR1 (Díaz-Martínez et al., 2015). (Note that this was originally called the A motif in yeast Acm1 (Burton et al., 2011), where it binds to Cdh1 into a pocket that closely resembles the sequence in human Cdc20. See Supplemental Experimental Procedures and Figures S3A–S3C.) But we found that the ABBA^{528} motif in BubR1 plays only a minor role in the checkpoint (Di Fiore et al., 2015).

 © 2016 The Authors. Published by Elsevier Inc.
This is an open access article under the CC BY license (http://creativecommons.org/licenses/by/4.0/).

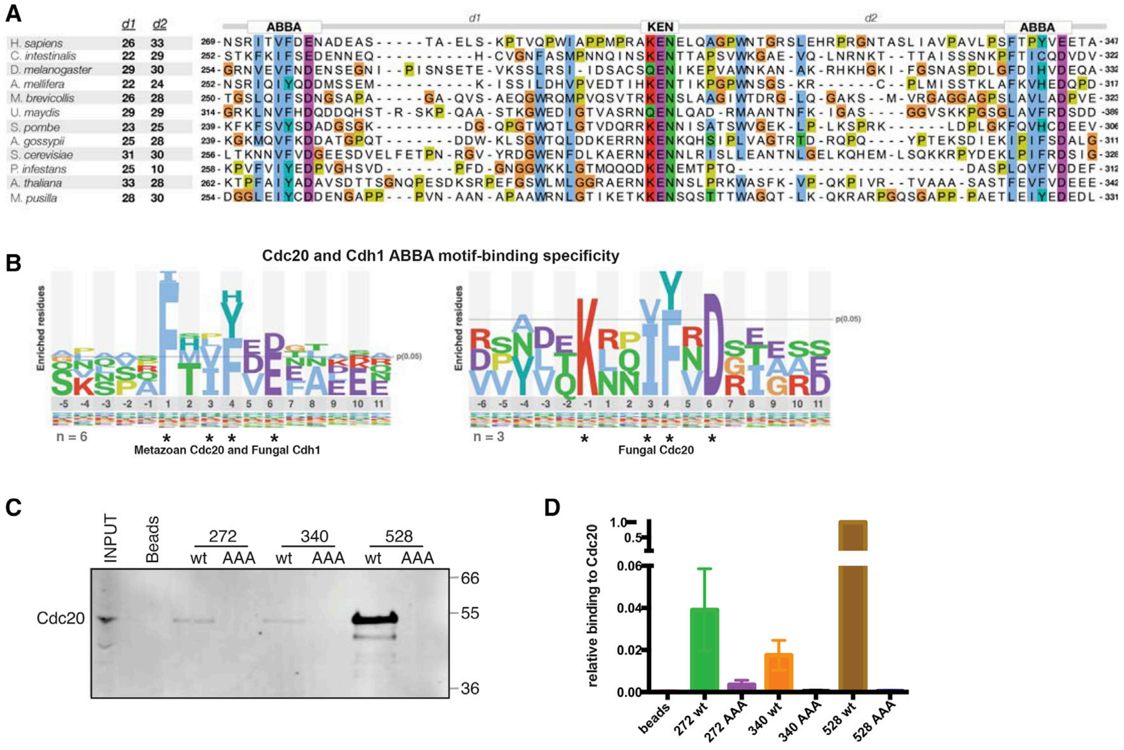

Figure 1. BubR1 ABBA Motifs Bind Cdc20

(A) Alignment of the central region of the MAD3/BUBR1-like proteins showing an ABBA-KEN-ABBA cassette conserved across the majority of eukaryotic kingdoms. The inter-motif spacing d1 (ABBA^{272}-KEN) and d2 (KEN-ABBA^{340}) are also shown.

(B) Relative binomial logos created from the experimentally characterized ABBA motif instances and their flanking residues. Logos show the $\log^{-10}$ of the binomial probability (see Supplemental Experimental Procedures for details). Asterisks signify positions that show strong preferences for a particular amino acid or chemically similar grouping of amino acids (e.g., hydrophobic, aromatic, or acidic positions). Logos are split to emphasize the divergence of the specificity of the fungal Cdc20 from the metazoan Cdc20 and fungal Cdh1. See Supplemental Experimental Procedures for details.

(C) Biotin-labeled peptides encompassing the BubR1 ABBA motifs were incubated with extract from HeLa cells synchronized in metaphase. Peptides in which positions 3, 4, and 6 of the consensus ABBA motif were mutated to alanine (AAA) were included as controls.

(D) Cdc20 binding shown in (C) was quantified and the mean and SEM from three independent experiments are shown. See also Figure S1.

Here we report the identification of two ABBA motifs in the N-terminal half of BubR1. We show that these motifs flank a KEN box to form a cassette that is highly conserved through evolution and is essential for the SAC because it is required for the MCC to bind and inhibit APC/C-Cdc20. Our results help to explain how the MCC rapidly inactivates the active APC/C in mitosis.

RESULTS

Two Additional BubR1 ABBA Motifs Bind Cdc20

The original metazoan ABBA motif instances matched a consensus of Fx[ILV][FHY]x[DE] (Di Fiore et al., 2015). However, we noticed that there were two highly conserved, but non-canonical, ABBA motifs in the N-terminal half of BubR1 (Figure 1A), starting at residues 272 and 340 (see Supplemental Experimental Procedures for the bioinformatics analysis). These instances, one of which does not have a consensus phenylalanine residue (Figure 1A), indicate that the motif consensus is more degenerate than previously thought (Figure 1B); therefore, we prefer the "ABBA motif" name over "Phe box." Peptides of ABBA^{272} and ABBA^{340} motifs could bind Cdc20 in metaphase HeLa cell extracts (Figures 1C and 1D), although more weakly than the ABBA^{528} peptide (Figure 1C), perhaps as a result of their non-canonical sequences. Control peptides in which alanine was substituted at conserved positions 3, 4, and 6 of the motif did not bind Cdc20. All three ABBA motifs appear to bind to the same site on Cdc20 because mutating residues in the ABBA binding pocket between blades 2 and 3 of the WD40 domain—either Y179E/I280Q (Di Fiore et al., 2015) or R262S (Díaz-Martínez et al., 2015)—reduced binding to all three peptides (Figures S1A and S1B).

ABBA Motifs Are Required for the MCC to Bind and Inhibit APC/C-Cdc20

Although the C-terminal ABBA^{528} motif is not required to form the MCC (Di Fiore et al., 2015; Díaz-Martínez et al., 2015; Kaisari et al., 2016), since ABBA^{272} and ABBA^{340} are in the N-terminal half of BubR1, we asked whether they are required to form a functional MCC. We used baculovirus-infected Sf9 cells to co-express His_6-Mad2, Cdc20, and Bub3 with streptavidin binding protein (SBP)-tagged BubR1 carrying inactivating mutations in

either the ABBA^{272} or the ABBA^{340} motif. Both mutants were incorporated into recombinant MCC (rMCC) in the same manner as wild-type (WT) BubR1 (Figure S1C), but when we assayed the ability of the rMCC to inhibit APC/C-Cdc20, neither mutant form of rMCC could inhibit active APC/C in an in vitro ubiquitylation assay (Figures 2A and 2B). In this, they strongly resembled the BubR1 D-box mutant that cannot act as a pseudo-substrate inhibitor (Izawa and Pines, 2015; Figures 2A and 2B); therefore, we assayed whether the ABBA^{272} and ABBA^{340} motifs were needed for the MCC to recognize APC/C-Cdc20.

We generated stable cell lines expressing small interfering RNA (siRNA)-resistant forms of FLAG-mRuby-tagged wild-type BubR1 or the ABBA^{272} or ABBA^{340} mutants from a tetracycline-inducible promoter. We depleted endogenous BubR1 and induced expression of the BubR1-ABBA mutants to physiological levels (Figure 3A). As controls, we assayed stable cell lines expressing BubR1 with inactivating mutations in the N-terminal KEN1 box at position 26, through which BubR1 binds Cdc20 within the core MCC (Burton and Solomon, 2007; Chao et al., 2012; King et al., 2007), or the D-box at position 224 or the KEN2 box at position 305 (between ABBA^{272} and ABBA^{340} motifs) that we previously showed are required to bind a second molecule of Cdc20 (Izawa and Pines, 2015). After enriching cells in prometaphase, we immunoprecipitated BubR1 and probed for MCC and APC/C components (Figures 2C and 2D; Figures S1G and S1H). As expected, the KEN1 mutant could not form the MCC, whereas the D-box, KEN2, ABBA^{272}, and ABBA^{340} mutants could all be incorporated into the core MCC, although the levels of Mad2 co-immunoprecipitating with these mutants were reduced compared to wild-type, potentially indicating a difference in the efficiency of MCC assembly or stability (Figures 2C and 2D). The most striking difference, however, was in the amount of co-immunoprecipitated APC/C: the D-box, KEN2, and ABBA^{340} mutations markedly reduced the level of APC/C compared to wild-type, and APC/C binding was eliminated for the ABBA^{272} mutant (Figures 2C and 2D). We conclude that ABBA^{272} in particular is essential for the MCC to bind to the APC/C.

ABBA Motifs Are Essential to Maintain the SAC In Vivo

We predicted that the ABBA^{272} and ABBA^{340} motifs would both be important for the SAC since they perturb MCC binding to the APC/C. To test this, we depleted endogenous BubR1 by siRNA and compared the ability of the D-box, KEN1, KEN2, ABBA^{272}, and ABBA^{340} mutants to wild-type BubR1 to restore the SAC in two ways: by measuring the time taken from nuclear envelope breakdown (NEBD) to anaphase—which is set by the SAC—and by measuring how long cells could remain arrested in mitosis in the presence of spindle poisons. In both assays, the KEN1 mutant was the most impaired since the MCC cannot form, but the ABBA^{272} mutant was also profoundly impaired, to a greater extent even than the D-box and KEN2 mutants (Figures 3B and 3C). The ABBA^{340} mutant had a slightly less severe phenotype, but cells were still accelerated through prometaphase and unable to sustain a mitotic arrest in the presence of nocodazole (Figures 3B and 3C). Similar results were observed in the RPE1-hTERT untransformed cell line, where the ABBA^{272} mutant also showed a more severe phenotype than ABBA^{340} (Figure S2A). Note that unlike the ABBA^{528} motif, the ABBA^{272} and ABBA^{340} motifs were not required to recruit Cdc20 to kinetochores (Figures S2B and S2C). In agreement with these results, the Cdc20 mutants that were unable to bind the ABBA motifs were also unable to maintain a mitotic arrest (Figures S2D and S2E) in HeLa cells. Thus, we conclude that the ABBA^{272} and ABBA^{340} motifs are essential for the SAC by mediating MCC binding and inhibition of the APC/C.

ABBA Motifs Are Part of a Conserved Cassette Important for the SAC

Evolutionary analysis revealed a further aspect of the importance of ABBA^{272} and ABBA^{340} for the function of BubR1. ABBA^{272} and ABBA^{340} flank the similarly highly conserved KEN2 box that is required for the pseudo-substrate inhibitor role of BubR1 (Izawa and Pines, 2015). This ABBA-KEN-ABBA cassette is conserved across almost all major eukaryotic supergroups (including Opisthokonta, Amoebozoa, Plantae, Chromalveolata, and Excavata) (Figure 4A). The conservation of the cassette for over a billion years of evolution points to an important functional role—especially given the relative transience of most short linear motifs over long evolutionary timescales (Davey et al., 2012). Furthermore, the ABBA-KEN-ABBA cassette is always found associated with the N-terminal KEN box, as clearly seen in the subfunctionalization of BubR1 and Bub1. BubR1 and Bub1 are the result of the duplication of a single multifunctional ancestral protein (Suijkerbuijk et al., 2012). The single Bub1-like proteins in simple chordates, e.g., *Ciona intestinalis*, reveal the likely architecture of the common ancestor of human Bub1 and BubR1 (Figure 4A). After the duplication of the ancestral Bub1-like protein, BubR1 and Bub1 diverged and took on specific roles. This happened on multiple occasions, and tracking the gain and loss of functional modules shows that the evolutionary path of sub-functionalization is remarkably predictable (Figures 4B and 4C). In nearly all cases, the BubR1-like proteins lost their MAD1 binding region and kinase domain (Figure 4D; Figure S3), whereas the Bub1-like proteins lost their N-terminal KEN box and ABBA-KEN-ABBA cassette (Figure 4E; Figure S4).

We noted that conservation of the cassette is always as a complete module in which the inter-motif distance is constrained despite the large evolutionary distances and high rates of mutation/insertion/deletion in the inter-motif regions (Figure 1A). This indicated that the spacing between the motifs might be important to interact with the correct surfaces on APC/C-Cdc20. If the ABBA^{272} motif and the KEN2 box bind to the APC/C-bound Cdc20 (for clarity we refer to this as Cdc20$^{APC/C}$), then modeling this onto the structure of Cdc20 predicts that reducing the spacing would preclude both motifs binding to their respective receptors. (The ABBA motif receptor is between blades 2 and 3 of the WD40 domain, and the KEN box binds the top surface of Cdc20.) To test these predictions, we deleted 12 amino acids between ABBA^{272} and the KEN2 box (Δ280-292) and 17 amino acids between the KEN2 box and ABBA^{340} (Δ319-336); siRNA and rescue experiments showed that neither mutant could function in the SAC (Figure 4F). To determine whether there were any important motifs in these linker regions, we restored the distance between both motifs with a Gly-Gly-Thr linker and found that this restored the checkpoint (Figure 4G), indicating that simply the

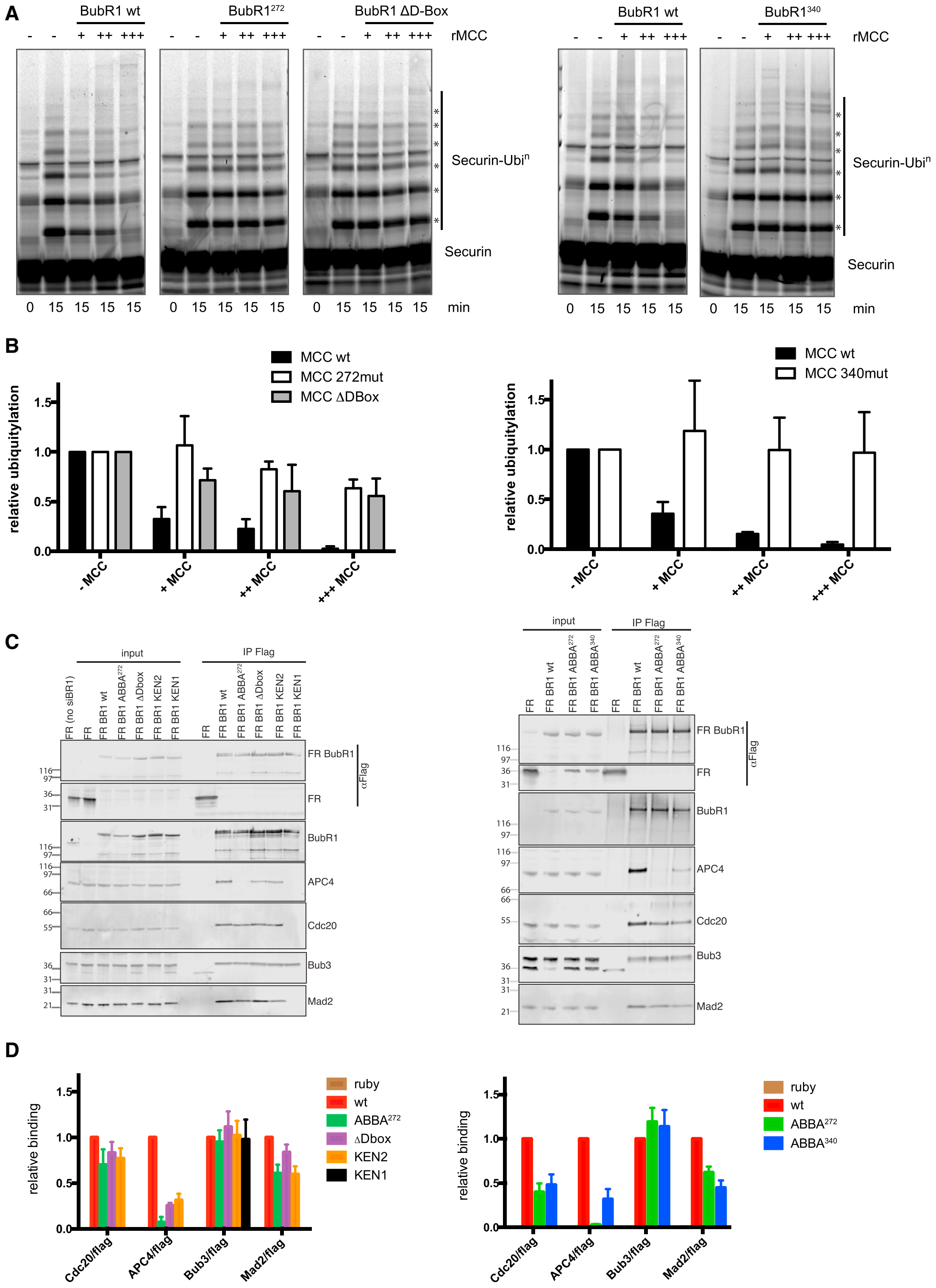

Figure 2. BubR1 ABBA²⁷² and ABBA³⁴⁰ Are Essential for the MCC to Bind and Inhibit the APC/C

(A) Apo-APC/C immunoprecipitated from Cdc20-depleted mitotic HeLa cell extracts was incubated with recombinant SBP-Cdc20 and increasing amounts of recombinant MCC (rMCC) containing wild-type, ABBA^{272} mutant, or ΔD-box mutant of BubR1 (left) or WT or ABBA^{340} mutant (right). Results shown are representative of three independent experiments.

(legend continued on next page)

correct spacing between the ABBA-KEN-ABBA motifs was important for SAC activity.

This led us to conceive a model in which the two ABBA motifs in the cassette stabilize the MCC onto the APC/C by binding the two Cdc20 molecules in the complex. In our model, the ABBA^{272} motif would bind to Cdc20$^{APC/C}$ and ABBA^{340} to Cdc20^{MCC} in the core MCC; therefore, ABBA^{272} would be essential for the MCC to bind the APC/C, but mutations in ABBA^{340} might be compensated by stabilizing the MCC. To test this, we stabilized the binding between Cdc20 and Mad2 in the MCC by co-expressing GBP (GFP binding protein)-Cdc20 and Venus-Mad2 in stable cell lines expressing either WT or ABBA mutant BubR1 and depleting endogenous BubR1 by siRNA (Izawa and Pines, 2015). In this system, stabilizing the MCC promotes mitotic arrest even in the absence of microtubule poisons (Izawa and Pines, 2015). Consistent with our prediction, we found that the stabilized MCC still required ABBA^{272} to arrest cells in mitosis and bind the APC/C, but that ABBA^{340} was no longer essential (Figures 4H–4J).

DISCUSSION

Here we have identified a conserved cassette in the N-terminal half of BubR1 that plays an essential role in the spindle assembly checkpoint. It contains two ABBA motifs flanking the previously characterized KEN2 box (Burton and Solomon, 2007; Elowe et al., 2010; King et al., 2007; Lara-Gonzalez et al., 2011). Both ABBA motifs bind Cdc20 and, although they are not required for the formation of the MCC, they are essential for the MCC to bind and inhibit active APC/C-Cdc20. Our data are consistent with a model in which ABBA^{272} binds to Cdc20$^{APC/C}$ and ABBA^{340} binds to Cdc20^{MCC}.

The ABBA-KEN-ABBA cassette is conserved in all kingdoms and this taxonomic range (eukaryota) is much larger than the previously characterized ABBA motif at position 528 in BubR1 (metazoa) that is also found in Cyclin A (metazoa), Bub1 (metazoa), Clb5 (saccharomycetales), and Acm1 (saccharomycetales). This indicates that the ABBA^{272} and ABBA^{340} motifs may be the original binding partners for the ABBA binding pocket on Cdc20. We note that *C. elegans* appears to be an exception to the conservation of the ABBA-KEN-ABBA cassette, and it will be interesting to determine whether the MCC binds stably to the APC/C through other motifs or whether the dynamics of MCC generation in worms makes stable binding of the MCC to the APC/C less crucial.

On at least five separate occasions during evolution, the N-terminal KEN1 box and the ABBA-KEN-ABBA cassette have been selected together in the BubR1-like proteins, whereas the MAD1 binding regions and kinase domains have been lost, and vice versa in the Bub1-like proteins. This indicates that the ABBA-KEN-ABBA cassette is functionally linked to the N-terminal pseudo-substrate KEN1 box motif. We hypothesize that evolutionary pressures drove the separation of the functionality of the ancestral protein into its substrate modification (kinase domain) plus MAD1 recruitment components, and its MCC inhibitory components (N-terminal KEN1 and the ABBA-KEN-ABBA cassette) to resolve adaptive conflicts between the modules in the ancestral protein (Hittinger and Carroll, 2007).

ABBA^{272} and ABBA^{340} specifically bind Cdc20 but with much lower affinity than the previously described ABBA^{528} (Di Fiore et al., 2015; Kaisari et al., 2016). Binding the MCC to the APC/C through these modular low-affinity binding sites would enable rapid association and dissociation kinetics, thereby allowing MCC binding to the APC/C to respond quickly to checkpoint activity: rapid MCC dissociation would activate the APC/C once the SAC was turned off, but newly produced MCC could quickly inactivate the APC/C should a kinetochore detach before anaphase.

We showed that MCC binds to a second molecule of Cdc20 as a pseudo-substrate inhibitor via the BubR1 D-box and KEN2 box (Izawa and Pines, 2015), but for this to be efficient, the MCC should prefer to bind to APC/C-associated Cdc20 over free Cdc20. Mutating the ABBA^{272} motif abolished binding to the APC/C and inactivated the SAC even though the MCC should still be able to bind Cdc20 as pseudo-substrate inhibitor (since the D-box and KEN2 box on BubR1 are still intact); thus, stable binding of the MCC onto the APC/C-Cdc20 is essential to inhibit the APC/C and arrest cells in mitosis. Mutating the ABBA^{340} motif reduces, but does not abolish, APC/C binding. We propose that the more severe phenotype of the ABBA^{272} mutant is likely to be because it synergizes with the D-box and KEN2 motifs to lock the MCC onto Cdc20$^{APC/C}$, and the ABBA^{340} motif further stabilizes the interaction between the MCC and the APC/C through binding between blades 2 and 3 of Cdc20^{MCC}. This would account for the conservation of the longer spacing between the KEN box and ABBA^{340} compared to ABBA^{272}.

Two structures of the MCC-APC/C complex were published while this study was under review (Alfieri et al., 2016; Yamaguchi et al., 2016). Both structures provide support for our proposal that the ABBA-KEN-ABBA cassette binds the MCC to the APC/C through binding the two Cdc20 molecules. In both structures, the ABBA^{272} and KEN2 motifs can be visualized binding to Cdc20$^{APC/C}$, but the peptide chain after this is not resolved. The authors of both studies suggested that ABBA^{528} in the C terminus of BubR1 might fold back to bind to Cdc20^{MCC}, but we previously showed that mutating ABBA^{528} has only a marginal effect on the SAC (Di Fiore et al., 2015). Instead, our data point to a much simpler solution: if ABBA^{272} binds to Cdc20$^{APC/C}$ and ABBA^{340} binds Cdc20^{MCC}, then the ABBA-KEN-ABBA cassette would lie across the MCC-APC/C structure as an almost linear chain (see Figure 2 in Alfieri et al., 2016).

(B) Ubiquitylation assays shown in (A) were quantified as shown in Figure S1D. Mean ± SD of three independent experiments is shown. Ratios between Cdc20 (MCC) and SBP-Cdc20 added to the reaction are quantified in Figures S1E and S1F.

(C) HeLa FRT/TO cell lines stably expressing inducible siRNA-resistant, FLAG-mRuby (FR)-BubR1 WT or mutants were transfected with siRNA to deplete endogenous BubR1. Anti-FLAG immunoprecipitations from nocodazole-arrested cells were analyzed by immunoblotting and visualized on a LI-COR Odyssey scanner.

(D) Mean ± SEM of protein levels of the experiments shown in (C) from four (left graph) or three (right graph) independent experiments. See also Figure S1.

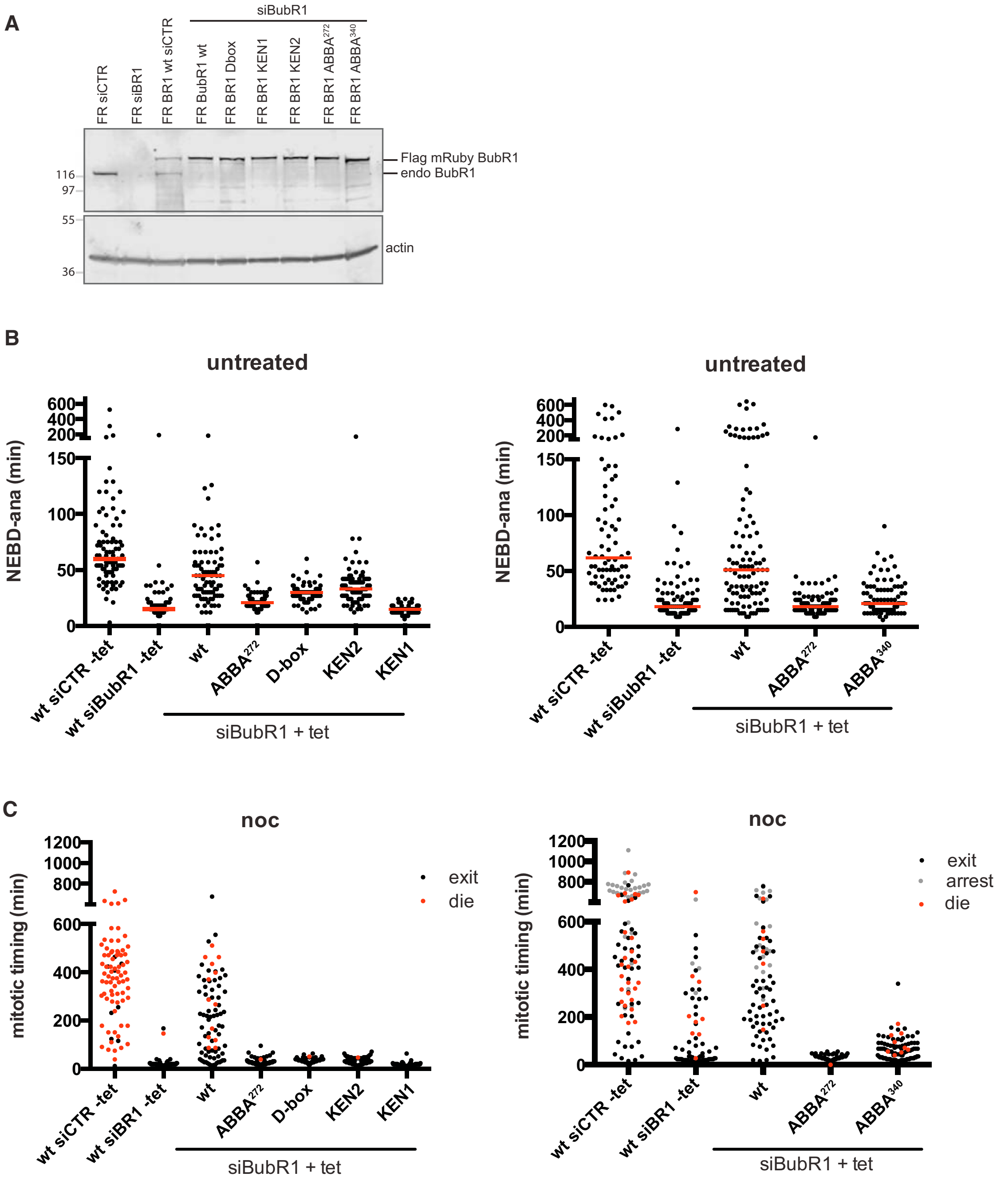

Figure 3. BubR1 ABBA^{272} and ABBA^{340} Are Essential for the SAC

(A) HeLa FRT/TO cell lines stably expressing inducible siRNA-resistant, FLAG-mRuby-tagged, wild-type or mutant BubR1 were transfected with control siRNA (siCTR) or siRNA against BubR1. Immunoblotting analysis shows the relative expression levels and the efficiency of depletion. Actin is a loading control.

(B) Mitotic timing of HeLa FRT/TO cell lines in (A) was measured with or without addition of tetracycline (tet). At least 80 cells per condition were analyzed. Results representative of three independent experiments.

(C) Experiments were performed as in (B) except that nocodazole (0.33 μM) was added at the beginning of the filming. At least 80 cells per condition were analyzed. Results representative of three independent experiments. See also Figure S2.

The structures of the MCC-APC/C complex show that Cdc20$^{APC/C}$ is tilted and rotated away from the position where it aligns with APC10 to form the bi-partite D-box receptor (Chao et al., 2012; da Fonseca et al., 2011; Herzog et al., 2009) and that BubR1 has additional contacts with APC2 that would inhibit the binding of an E2 (Alfieri et al., 2016; Yamaguchi et al., 2016). Thus, the MCC inhibits the APC/C in multiple ways, not just as a pseudo-substrate, which agrees with our

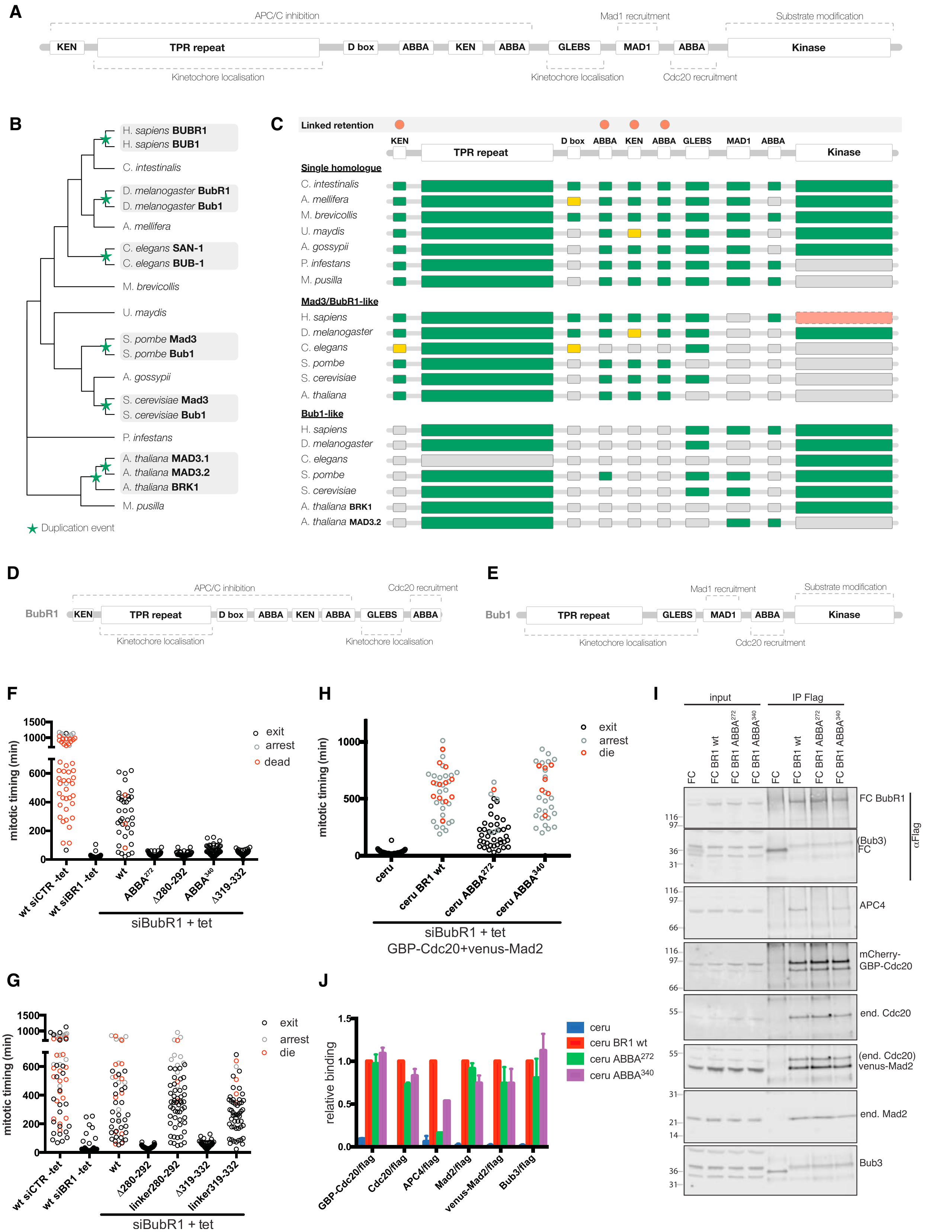

(legend on next page)

finding that the ABBA^{272} mutant phenotype is more severe than either the D-box or KEN2 box mutant.

Lastly, our identification of the ABBA motif-Cdc20 interaction as essential to the SAC unveils a potential new way to target the SAC specifically by using small molecules as an alternative to the protein kinase inhibitors currently being developed for clinical trials (Wengner et al., 2016).

EXPERIMENTAL PROCEDURES

Cell Culture and Synchronization

HeLa FRT/TO and RPE1-hTERT FRT/TO were transfected using the Flp-In system (Invitrogen) to generate stable cell lines. Cells were grown and synchronized as previously described (Di Fiore et al., 2015). Cells were treated with tetracycline (1 μg/mL, Calbiochem) 12 hr before harvesting. For prometaphase arrest, nocodazole (0.33 μM, Sigma) was added during release from a thymidine block, and 12 hr later, MG132 (10 μM, Calbiochem) was added and cells collected by mitotic shake-off after 2 hr. To obtain metaphase cells, we released cells arrested in prometaphase by dimethyl anastron (DMA) (10 μM, Calbiochem) into reversine (0.5 μM, Cayman Chemical) and MG132 for 2 hr.

Peptide Pull-Down and Competition

Biotin-conjugated peptides (Selleck Chemicals) were synthesized with the following sequences: wild-type 272 (QMQNNSRITVFDENADEAST), wild-type 340 (VPAVLPSFTPYVEETAQQPV), and wild-type 528 (SKGPSVPFSIF DEFLLSEKK). In the mutant peptides, the third, fourth, and sixth amino acids of the motif were mutated to alanine. Cells arrested in metaphase were lysed in PBS plus 0.5% Triton X-100 and protease inhibitor cocktail (Roche), and the extracts were cleared by centrifugation and were incubated with the biotinylated peptides coupled to streptavidin Ultralink beads (Pierce) for 1 hr at 4°C. For binding to FLAG-Cdc20 mutants, BSA 5 mg/mL was preincubated with the beads and added during the pull-down. After extensive washes, associated proteins were eluted at 65°C for 5 min, separated by SDS-PAGE, and analyzed by immunoblotting on a LI-COR Odyssey scanner.

Imaging and Analysis

For time-lapse microscopy, cells were seeded and transfected on an 8-well chamber slide (μslide, Ibidi) and the medium replaced with Leibovitz's L-15 medium (Invitrogen) just before filming. Differential interference contrast (DIC) and fluorescence images were captured every 5 min with a Nikon Eclipse Ti microscope equipped with a Hamamatsu Flash 4.0 sCMOS camera using micromanager software. DIC microscopy was used to monitor mitotic phases. To measure the intensity of kinetochore, we filmed localization cells on a spinning disk microscope (Intelligent Imaging Innovations). Maximum projections of single z sections at NEBD were obtained and the kinetochore intensity quantified using ImageJ.

Immunoprecipitation

Cells were lysed in 50 mM Tris-HCl (pH 7.8), 150 mM NaCl, 0.2% NP40, and 1 mM EDTA plus protease inhibitor cocktail (Roche) and microcystin (10 nM, LGC Promotech) for 20 min on ice and clarified at 12,000 × *g* for 15 min at 4°C. Complexes were immunoprecipitated for 1 hr at 4°C with anti-FLAG (M2, Sigma) covalently coupled to Dynabeads protein G (Invitrogen). After five washes in lysis buffer, proteins were eluted from beads by incubating 5 min at 65°C in SDS-PAGE sample buffer.

rMCC Purification and In Vitro Ubiquitylation Assays

MultiBac constructs encoding the sequences for Cdc20, SBP-BubR1 (WT or mutants), His_6-Mad2, and Bub3 were used to infect Sf9 insect cells. Recombinant MCC was purified by biotin elution from Strep Agarose Beads (Invitrogen) followed by Ni-NTA beads (QIAGEN). Purified rMCC was analyzed by silver staining (Sigma Aldrich) after SDS-PAGE. SBP-Cdc20 was expressed and purified from Sf9 cells. In vitro ubiquitylation assays were performed as described previously (Izawa and Pines, 2015), except that an APC4 monoclonal antibody was used to isolate the apo-APC/C. APC/C immunoprecipitated from Cdc20-depleted mitotic HeLa cell extract was pre-incubated on ice with rMCC and recombinant SBP-Cdc20. Securin labeled with fluorescent maleimide dyes (LI-COR) was used as a substrate. Reactions were started by the addition of E1 and E2 enzymes, ubiquitin and ATP, and incubated at 37°C for 15 min. Reaction products were separated by SDS-PAGE and visualized using a LI-COR Odyssey scanner. Levels of Cdc20 and other components were quantified by immunoblotting.

Figure 4. The ABBA^{272}-KEN2-ABBA^{340} Module Is Conserved through Evolution

(A) Predicted pre-duplication architecture of the ancestral protein to the human Bub1-like proteins—BubR1 and Bub1—based on *Ciona intestinalis* that has a single Bub1-like protein. The ancestral Bub1-like protein likely consisted of: an N-terminal KEN box pseudo-substrate domain; a TPR domain that stabilized the interaction between the N-terminal KEN and Cdc20 in the MCC (Chao et al., 2012) and bound to KNL1 to promote kinetochore recruitment (Krenn et al., 2012); a Bub3-binding motif (Wang et al., 2001); a MAD1 recruitment module (Klebig et al., 2009); an ABBA motif that promoted Cdc20 kinetochore recruitment (Di Fiore et al., 2015); and a C-terminal kinase domain (Bolanos-Garcia and Blundell, 2011; Suijkerbuijk et al., 2012). The region between the TPR domain and GLEBS motif also contained the putative ABBA motifs, a KEN box, and a D-box.

(B) An evolutionary tree of the Bub1-like proteins in selected eukaryotic species. The tree contains two distinct classes of species: (1) those where a duplication event has resulted in two or more Bub1-like proteins (light gray boxes) and (2) those where a single Bub1-like protein is present. The position of these classes in the tree was chosen to define points of Bub1-like protein duplication (green star).

(C) The modular architecture of the species in (B) grouped by (1) Bub1-like proteins from single homolog species, (2) BubR1-like proteins from multiple homolog species, and (3) Bub1-like proteins from multiple homolog species. Architecture shows the retained modules (green), inconclusively retained modules (yellow), retained modules characterized as non-functional (red), and potentially absent modules (gray). The modules that, post-duplication, have been simultaneously retained in BubR1 and lost in Bub1 independently on multiple occasions are marked above the architecture by red circles. Search details are shown in Table S1.

(D and E) Architecture of the functional modules in the human BubR1 (D) and human Bub1 (E) showing the role of the retained modules post-subfunctionalization. See Figure S3 and http://slim.ucd.ie/abbakenabba/ for alignments.

(F) HeLa FRT/TO cell lines stably expressing siRNA-resistant, FLAG-mRuby-tagged, WT or mutant BubR1 from a tetracycline (tet)-inducible promoter were transfected with control siRNA or siRNA against BubR1 (siBubR1), and nocodazole was added at the beginning of filming. Mitotic timing was measured with or without addition of tetracycline. The mitotic timing of WT BubR1 and ABBA^{272} or ABBA^{340} mutants was compared to deletion mutants in the inter-motif region. At least 50 cells per condition were analyzed. Results representative of three independent experiments.

(G) HeLa FRT/TO cell lines expressing the indicated BubR1 proteins were treated as in (F) and the mitotic timings compared. At least 50 cells per condition were analyzed. Results representative of three independent experiments.

(H) HeLa FRT/TO cell lines stably expressing siRNA-resistant, FLAG-Cerulean (FC) BubR1 WT or mutant from a tetracycline-inducible promoter were transfected with siRNA against BubR1 together with plasmids expressing mCherry-GBP-Cdc20 and Venus-Mad2. Mitotic timing was measured after addition of tetracycline. At least 35 cells per condition were analyzed. Results are representative of three independent experiments.

(I) HeLa FRT/TO cell lines stably expressing inducible siRNA-resistant, FLAG-Cerulean-BubR1 WT or mutants were transfected as for (H), and anti-FLAG immunoprecipitations from nocodazole-arrested cells were analyzed by immunoblotting and visualized on a LI-COR Odyssey scanner.

(J) The mean and SEM of protein levels from two independent experiments in (I). A third experiment was consistent with these results, but the siRNA did not deplete endogenous BubR1 to the same extent so these results were not included in the calculations.

Sequence and Evolutionary Analysis

Bub1-like proteins were retrieved from 13 eukaryotic organisms: *H. sapiens*, *C. intestinalis*, *D. melanogaster*, *A. mellifera*, *C. elegans*, *M. brevicollis*, *U. maydis*, *S. pombe*, *A. gossypii*, *S. cerevisiae*, *P. infestans*, *A. thaliana*, and *M. pusilla*. Species were chosen based on the Bub1-like protein family tree constructed by Suijkerbuijk et al. (2012) to create subclades of two species: one containing a single copy Bub1-like protein and one containing two or more Bub1-like proteins. Proteins were split into three groups: (1) single copy Bub1-like proteins, (2) duplicated Bub1-like proteins, and (3) duplicated BubR1-like proteins. The retained modules, inconclusively retained modules, and potentially absent modules in Bub1-like proteins were defined as described in Supplemental Experimental Procedures. Absent motifs could not be detected by the search criteria but may still be present; for example, the structure of the D-box binding pocket of *S. pombe* Cdc20 is occupied by a non-consensus peptide from Mad3 (Chao et al., 2012).

SUPPLEMENTAL INFORMATION

Supplemental Information includes Supplemental Experimental Procedures, three figures, and one table and can be found with this article online at http://dx.doi.org/10.1016/j.molcel.2016.11.006.

AUTHOR CONTRIBUTIONS

B.D.F., C.W., and J.P. designed the experiments. B.D.F. and C.W. performed the experiments. N.E.D. performed the sequence and evolutionary analysis. All authors contributed to the analysis of the results. B.D.F., N.E.D., and J.P. wrote the paper.

ACKNOWLEDGMENTS

We are grateful to Jörg Mansfeld and Daisuke Izawa for help with preliminary results and discussions and to Toby Gibson for discussions and helpful comments. We thank David Barford and Claudio Alfieri for sharing unpublished results. We are grateful to Mark Solomon for agreeing to change the name of the A motif to the ABBA motif. We are grateful to everyone in our laboratory for discussions and critical reading of the manuscript. This work was supported by an SFI Starting Investigator Research Grant (13/SIRG/2193) to N.E.D. and a CR UK Programme grant C29/A13678 to J.P. J.P. acknowledges the financial support of Wellcome Trust Grant 092096 and CR UK Grant C6946/A14492 core support to the Gurdon Institute.

Received: June 17, 2016
Revised: September 12, 2016
Accepted: October 31, 2016
Published: December 8, 2016

REFERENCES

Alfieri, C., Chang, L., Zhang, Z., Yang, J., Maslen, S., Skehel, M., and Barford, D. (2016). Molecular basis of APC/C regulation by the spindle assembly checkpoint. Nature *536*, 431–436.

Bolanos-Garcia, V.M., and Blundell, T.L. (2011). BUB1 and BUBR1: multifaceted kinases of the cell cycle. Trends Biochem. Sci. *36*, 141–150.

Burton, J.L., and Solomon, M.J. (2007). Mad3p, a pseudosubstrate inhibitor of APCCdc20 in the spindle assembly checkpoint. Genes Dev. *21*, 655–667.

Burton, J.L., Xiong, Y., and Solomon, M.J. (2011). Mechanisms of pseudosubstrate inhibition of the anaphase promoting complex by Acm1. EMBO J. *30*, 1818–1829.

Chao, W.C., Kulkarni, K., Zhang, Z., Kong, E.H., and Barford, D. (2012). Structure of the mitotic checkpoint complex. Nature *484*, 208–213.

Clute, P., and Pines, J. (1999). Temporal and spatial control of cyclin B1 destruction in metaphase. Nat. Cell Biol. *1*, 82–87.

da Fonseca, P.C.A., Kong, E.H., Zhang, Z., Schreiber, A., Williams, M.A., Morris, E.P., and Barford, D. (2011). Structures of APC/C^{Cdh1} with substrates identify Cdh1 and Apc10 as the D-box co-receptor. Nature *470*, 274–278.

Davey, N.E., Van Roey, K., Weatheritt, R.J., Toedt, G., Uyar, B., Altenberg, B., Budd, A., Diella, F., Dinkel, H., and Gibson, T.J. (2012). Attributes of short linear motifs. Mol. Biosyst. *8*, 268–281.

De Antoni, A., Pearson, C.G., Cimini, D., Canman, J.C., Sala, V., Nezi, L., Mapelli, M., Sironi, L., Faretta, M., Salmon, E.D., and Musacchio, A. (2005). The Mad1/Mad2 complex as a template for Mad2 activation in the spindle assembly checkpoint. Curr. Biol. *15*, 214–225.

Di Fiore, B., Davey, N.E., Hagting, A., Izawa, D., Mansfeld, J., Gibson, T.J., and Pines, J. (2015). The ABBA motif binds APC/C activators and is shared by APC/C substrates and regulators. Dev. Cell *32*, 358–372.

Díaz-Martínez, L.A., Tian, W., Li, B., Warrington, R., Jia, L., Brautigam, C.A., Luo, X., and Yu, H. (2015). The Cdc20-binding Phe box of the spindle checkpoint protein BubR1 maintains the mitotic checkpoint complex during mitosis. J. Biol. Chem. *290*, 2431–2443.

Dick, A.E., and Gerlich, D.W. (2013). Kinetic framework of spindle assembly checkpoint signalling. Nat. Cell Biol. *15*, 1370–1377.

Elowe, S., Dulla, K., Uldschmid, A., Li, X., Dou, Z., and Nigg, E.A. (2010). Uncoupling of the spindle-checkpoint and chromosome-congression functions of BubR1. J. Cell Sci. *123*, 84–94.

Fang, G., Yu, H., and Kirschner, M.W. (1998). The checkpoint protein MAD2 and the mitotic regulator CDC20 form a ternary complex with the anaphase-promoting complex to control anaphase initiation. Genes Dev. *12*, 1871–1883.

Han, J.S., Vitre, B., Fachinetti, D., and Cleveland, D.W. (2014). Bimodal activation of BubR1 by Bub3 sustains mitotic checkpoint signaling. Proc. Natl. Acad. Sci. USA *111*, E4185–E4193.

Hardwick, K.G., Johnston, R.C., Smith, D.L., and Murray, A.W. (2000). MAD3 encodes a novel component of the spindle checkpoint which interacts with Bub3p, Cdc20p, and Mad2p. J. Cell Biol. *148*, 871–882.

Hein, J.B., and Nilsson, J. (2014). Stable MCC binding to the APC/C is required for a functional spindle assembly checkpoint. EMBO Rep. *15*, 264–272.

Herzog, F., Primorac, I., Dube, P., Lenart, P., Sander, B., Mechtler, K., Stark, H., and Peters, J.M. (2009). Structure of the anaphase-promoting complex/cyclosome interacting with a Mitotic Checkpoint Complex. Science *323*, 1477–1481.

Hittinger, C.T., and Carroll, S.B. (2007). Gene duplication and the adaptive evolution of a classic genetic switch. Nature *449*, 677–681.

Hwang, L.H., Lau, L.F., Smith, D.L., Mistrot, C.A., Hardwick, K.G., Hwang, E.S., Amon, A., and Murray, A.W. (1998). Budding yeast Cdc20: a target of the spindle checkpoint. Science *279*, 1041–1044.

Izawa, D., and Pines, J. (2012). Mad2 and the APC/C compete for the same site on Cdc20 to ensure proper chromosome segregation. J. Cell Biol. *199*, 27–37.

Izawa, D., and Pines, J. (2015). The mitotic checkpoint complex binds a second CDC20 to inhibit active APC/C. Nature *517*, 631–634.

Kaisari, S., Sitry-Shevah, D., Miniowitz-Shemtov, S., and Hershko, A. (2016). Intermediates in the assembly of mitotic checkpoint complexes and their role in the regulation of the anaphase-promoting complex. Proc. Natl. Acad. Sci. USA *113*, 966–971.

Kim, S.H., Lin, D.P., Matsumoto, S., Kitazono, A., and Matsumoto, T. (1998). Fission yeast Slp1: an effector of the Mad2-dependent spindle checkpoint. Science *279*, 1045–1047.

King, E.M., van der Sar, S.J., and Hardwick, K.G. (2007). Mad3 KEN boxes mediate both Cdc20 and Mad3 turnover, and are critical for the spindle checkpoint. PLoS ONE *2*, e342.

Klebig, C., Korinth, D., and Meraldi, P. (2009). Bub1 regulates chromosome segregation in a kinetochore-independent manner. J. Cell Biol. *185*, 841–858.

Krenn, V., Wehenkel, A., Li, X., Santaguida, S., and Musacchio, A. (2012). Structural analysis reveals features of the spindle checkpoint kinase Bub1-kinetochore subunit Knl1 interaction. J. Cell Biol. *196*, 451–467.

Lara-Gonzalez, P., Scott, M.I., Diez, M., Sen, O., and Taylor, S.S. (2011). BubR1 blocks substrate recruitment to the APC/C in a KEN-box-dependent manner. J. Cell Sci. *124*, 4332–4345.

Lischetti, T., Zhang, G., Sedgwick, G.G., Bolanos-Garcia, V.M., and Nilsson, J. (2014). The internal Cdc20 binding site in BubR1 facilitates both spindle assembly checkpoint signalling and silencing. Nat. Commun. *5*, 5563.

Luo, X., Tang, Z., Rizo, J., and Yu, H. (2002). The Mad2 spindle checkpoint protein undergoes similar major conformational changes upon binding to either Mad1 or Cdc20. Mol. Cell *9*, 59–71.

Morrow, C.J., Tighe, A., Johnson, V.L., Scott, M.I., Ditchfield, C., and Taylor, S.S. (2005). Bub1 and aurora B cooperate to maintain BubR1-mediated inhibition of APC/CCdc20. J. Cell Sci. *118*, 3639–3652.

Nilsson, J., Yekezare, M., Minshull, J., and Pines, J. (2008). The APC/C maintains the spindle assembly checkpoint by targeting Cdc20 for destruction. Nat. Cell Biol. *10*, 1411–1420.

Primorac, I., and Musacchio, A. (2013). Panta rhei: the APC/C at steady state. J. Cell Biol. *201*, 177–189.

Sironi, L., Mapelli, M., Knapp, S., De Antoni, A., Jeang, K.T., and Musacchio, A. (2002). Crystal structure of the tetrameric Mad1-Mad2 core complex: implications of a 'safety belt' binding mechanism for the spindle checkpoint. EMBO J. *21*, 2496–2506.

Sudakin, V., Chan, G.K., and Yen, T.J. (2001). Checkpoint inhibition of the APC/C in HeLa cells is mediated by a complex of BUBR1, BUB3, CDC20, and MAD2. J. Cell Biol. *154*, 925–936.

Suijkerbuijk, S.J., van Dam, T.J., Karagöz, G.E., von Castelmur, E., Hubner, N.C., Duarte, A.M., Vleugel, M., Perrakis, A., Rüdiger, S.G., Snel, B., and Kops, G.J. (2012). The vertebrate mitotic checkpoint protein BUBR1 is an unusual pseudokinase. Dev. Cell *22*, 1321–1329.

Wang, X., Babu, J.R., Harden, J.M., Jablonski, S.A., Gazi, M.H., Lingle, W.L., de Groen, P.C., Yen, T.J., and van Deursen, J.M.A. (2001). The Mitotic Checkpoint Protein hBUB3 and the mRNA export factor hRAE1 interact with GLE2p-binding sequence (GLEBS)-containing proteins. J. Biol. Chem. *276*, 26559–26567.

Wengner, A.M., Siemeister, G., Koppitz, M., Schulze, V., Kosemund, D., Klar, U., Stoeckigt, D., Neuhaus, R., Lienau, P., Bader, B., et al. (2016). Novel Mps1 kinase inhibitors with potent antitumor activity. Mol. Cancer Ther. *15*, 583–592.

Yamaguchi, M., VanderLinden, R., Weissmann, F., Qiao, R., Dube, P., Brown, N.G., Haselbach, D., Zhang, W., Sidhu, S.S., Peters, J.-M., et al. (2016). Cryo-EM of Mitotic Checkpoint Complex-bound APC/C reveals reciprocal and conformational regulation of ubiquitin ligation. Mol. Cell *63*, 593–607.

Zhang, Y., and Lees, E. (2001). Identification of an overlapping binding domain on Cdc20 for Mad2 and anaphase-promoting complex: model for spindle checkpoint regulation. Mol. Cell. Biol. *21*, 5190–5199.

Molecular Cell, Volume *64*

Supplemental Information

The Mitotic Checkpoint Complex
Requires an Evolutionary Conserved Cassette
to Bind and Inhibit Active APC/C

Barbara Di Fiore, Claudia Wurzenberger, Norman E. Davey, and Jonathon Pines

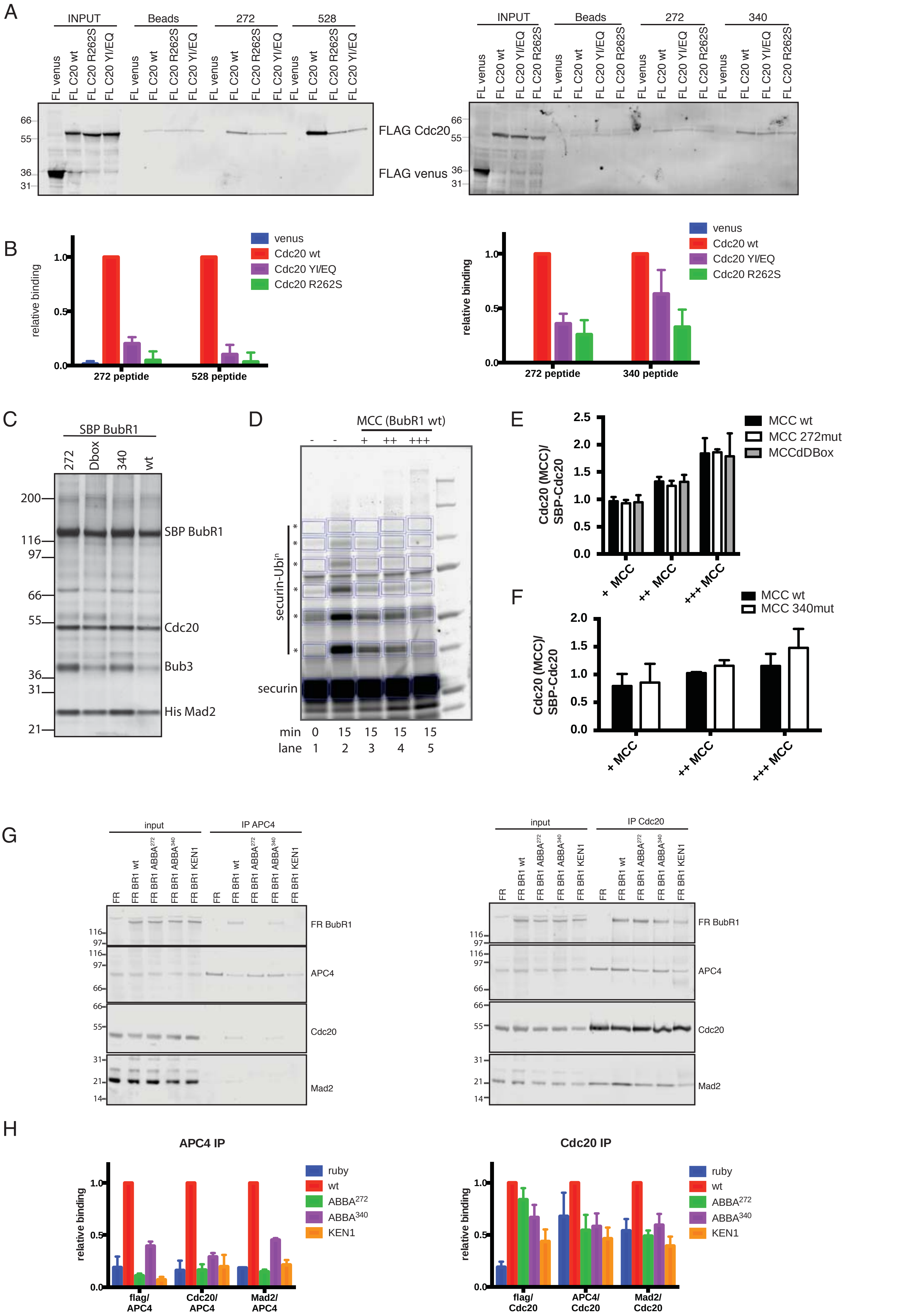

A
INPUT
Beads
272
528
340
FL venus
FL C20 wt
FL C20 R262S
FL C20 YI/EQ
FLAG Cdc20
FLAG venus
B
relative binding
272 peptide
528 peptide
340 peptide
venus
Cdc20 wt
Cdc20 YI/EQ
Cdc20 R262S
C
SBP BubR1
272
Dbox
340
wt
Cdc20
Bub3
His Mad2
D
MCC (BubR1 wt)
securin-Ubi^n
securin
min
lane
E
Cdc20 (MCC)/ SBP-Cdc20
MCC wt
MCC 272mut
MCCdDBox
+ MCC
++ MCC
+++ MCC
F
MCC wt
MCC 340mut
G
input
IP APC4
IP Cdc20
FR
FR BR1 wt
FR BR1 ABBA^{272}
FR BR1 ABBA^{340}
FR BR1 KEN1
FR BubR1
APC4
Cdc20
Mad2
H
APC4 IP
Cdc20 IP
ruby
wt
ABBA^{272}
ABBA^{340}
KEN1
flag/ APC4
Cdc20/ APC4
Mad2/ APC4
flag/ Cdc20
APC4/ Cdc20
Mad2/ Cdc20

Figure S1

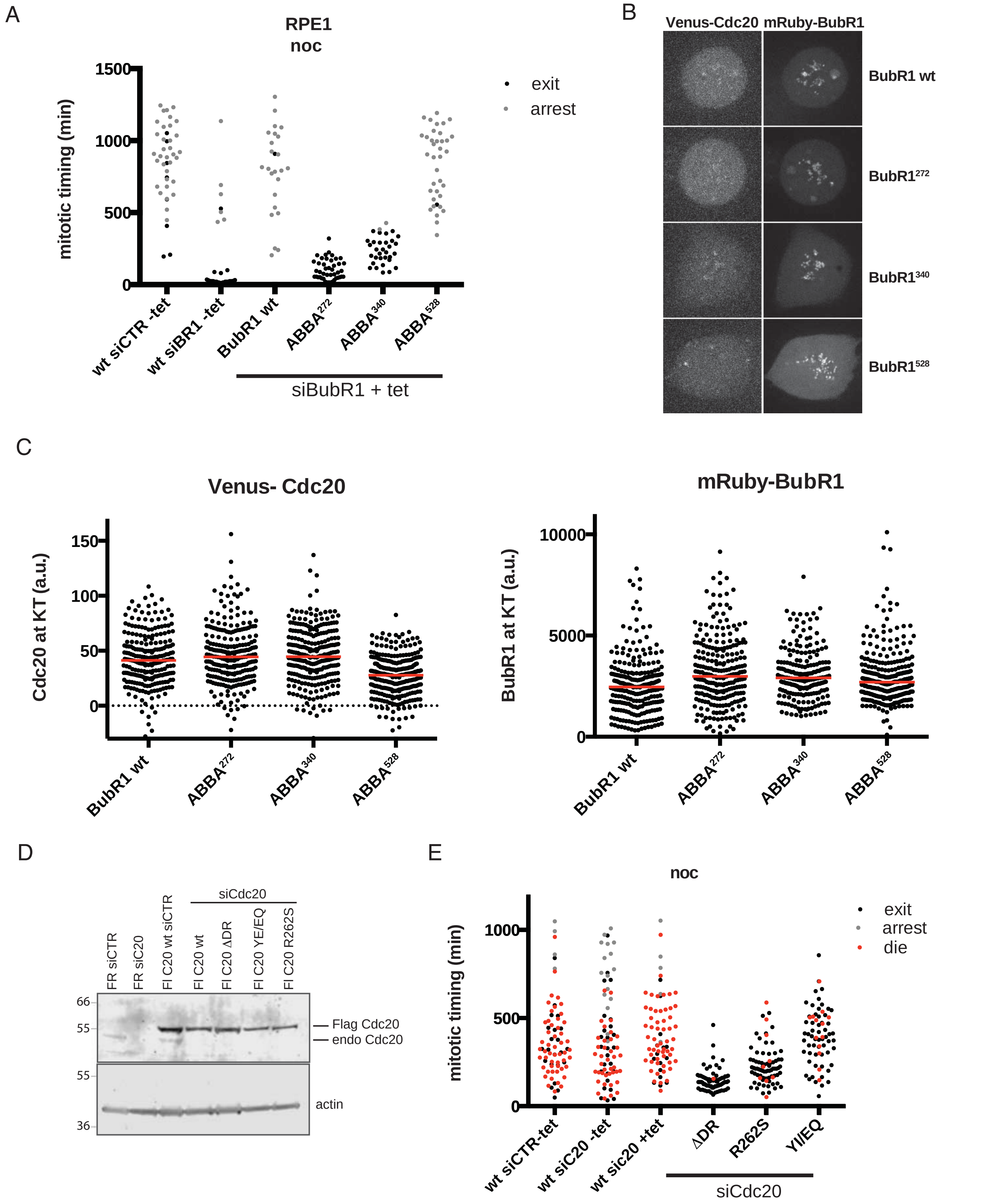

A
RPE1
noc
mitotic timing (min)
1500
1000
500
0
exit
arrest
wt siCTR -tet
wt siBR1 -tet
BubR1 wt
ABBA272
ABBA340
ABBA528
siBubR1 + tet
B
Venus-Cdc20
mRuby-BubR1
BubR1 wt
BubR1272
BubR1340
BubR1528
C
Venus- Cdc20
Cdc20 at KT (a.u.)
150
100
50
0
BubR1 wt
ABBA272
ABBA340
ABBA528
mRuby-BubR1
BubR1 at KT (a.u.)
10000
5000
0
D
FR siCTR
FR siC20
Fl C20 wt siCTR
siCdc20
Fl C20 wt
Fl C20 ΔDR
Fl C20 YE/EQ
Fl C20 R262S
66
55
55
36
Flag Cdc20
endo Cdc20
actin
E
noc
mitotic timing (min)
1000
500
0
exit
arrest
die
wt siCTR-tet
wt siC20 -tet
wt sic20 +tet
ΔDR
R262S
YI/EQ
siCdc20

Figure S2

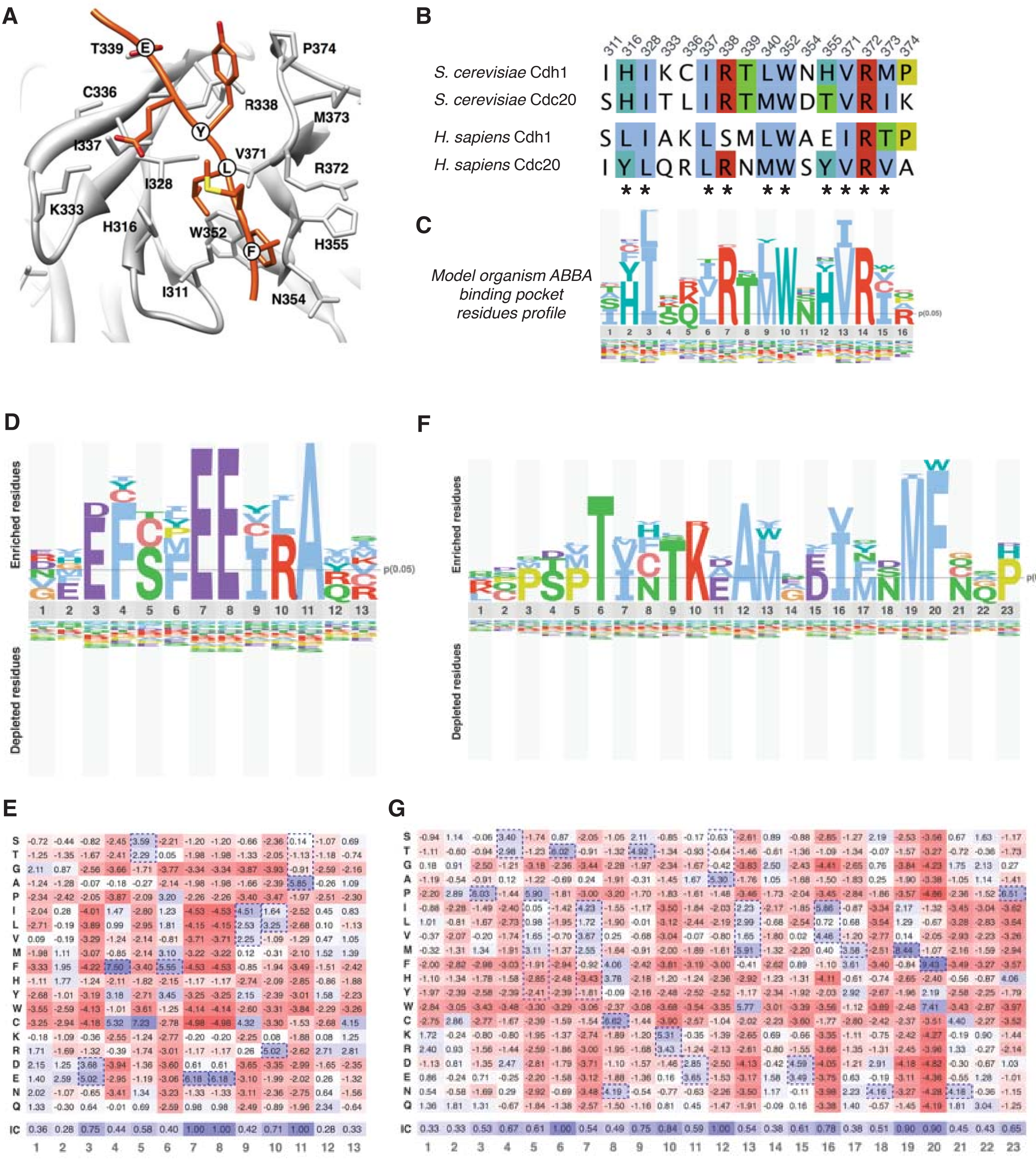
A
T339
P374
C336
R338
M373
I337
V371
R372
I328
K333
H316
W352
H355
I311
N354
B
S. cerevisiae Cdh1
S. cerevisiae Cdc20
H. sapiens Cdh1
H. sapiens Cdc20
C
Model organism ABBA binding pocket residues profile
D
Enriched residues
Depleted residues
F
Enriched residues
Depleted residues
E
G

Fig S3

Supplemental Figure legends

Supplemental Figure 1 - Related to Figure 1 and 2

(A) Peptide pull-downs were performed as in Fig1C, with extracts from HeLa FRT/TO cell lines expressing siRNA-resistant tetracyclin-inducible wild-type (wt) or mutant FLAG-tagged Cdc20 (FL C20). Endogenous Cdc20 was depleted by siRNA. (B) Quantification of the peptide pull-downs shown in panel A. Mean and SEM from 3 independent experiments. The reduction in binding of the mutant $ABBA^{340}$ to Cdc20 is more variable than mutant $ABBA^{272}$, potentially due to the presence of a non-canonical Pro at position 3 of $ABBA^{340}$, which might therefore have evolved other residues to stabilise the binding to Cdc20. (C) Silver stain of recombinant MCC containing wild type or mutant SBP-BubR1 purified from baculovirus-infected insect cells and analysed by SDS PAGE. (D) Ubiquitylation assay similar to Fig 2A is shown with the bands quantified for Fig 2B highlighted in blue. To calculate relative ubiquitylation, quantification was performed as follows: the fluorescence value of the bands in lane 1 were subtracted from each band of lane 2 to 5 migrating with the same mobility, and the sum of the 6 bands (marked with asterisks) designated as ubiquitylated securin. Relative ubiquitylation was calculated by dividing the values of lanes 3 to 5 by the value obtained in lane 2, considered to be the maximum ubiquitylation, in the absence of MCC. (E-F) Quantification of Cdc20 levels in the ubiquitylation reactions in Fig 2A as analysed by quantitative immunoblotting. (G) HeLa FRT/TO cell lines stably expressing inducible siRNA-resistant FLAG-mRuby (FR)-BubR1, wt or mutants, were transfected with siRNA to knockdown endogenous BubR1. Anti-APC4 (left) and anti-Cdc20 (right) immunoprecipitations from nocodazole-arrested cells were analysed by immunoblotting and visualised on a Li-COR Odyssey scanner. (H) Mean and SEM of protein levels from 4 independent experiments shown in panel G. Note that although the KEN1 BubR1 mutant is unable to form the MCC it can still bind Cdc20 through its $ABBA^{528}$ motif.

Supplemental Figure 2 - Related to Figure 3

(A) RPE1 FRT/TO cell lines stably expressing inducible siRNA-resistant FLAG-mRuby tagged BubR1, wild type or mutants, were transfected with siRNA against BubR1 or control siRNA (siCTR) and the mitotic timing measured in cells treated with nocodazole (0.33μM). At least 30 cells per condition were analysed. Results representative of 3 independent experiments. (B) Representative images of RPE1 FRT/TO cell lines expressing Cdc20 endogenously-tagged at one allele with the Venus fluorescent protein, and stably expressing siBubR1-resistant inducible FLAG-mRuby BubR1, wt or mutant, after depleting endogenous BubR1 with siRNA and treated with nocodazole (0.33μM). Images were acquired at the spinning disk with 10% laser power to reduce photo damage/fluorescence bleaching. (C) Kinetochore levels of Cdc20 were measured in cells shown in Fig S2B at the first time point after Nuclear Envelope Breakdown, selecting the kinetochores based on mRuby-BubR1 signal (right panel) and measuring the same area for Venus-Cdc20 images (left panel). At least 250 kinetochores from 15 cells were quantified for each condition. (D) HeLa FRT/TO cell lines stably expressing inducible siRNA-resistant FLAG-tagged Cdc20, wild type, D-box receptor (ΔDR) or ABBA receptor (R262S and YI/EQ) mutants, were transfected with siRNA against Cdc20 or control siRNA

(siCTR). FLAG mRuby (FR) cell lines are used as control. Immunoblotting analysis shows the expression levels of ectopic Cdc20 compared with endogenous Cdc20, and the efficiency of depletion. Actin is a loading control. (E) HeLa FRT/TO cells characterised in panel D were treated with nocodazole (0.33μM) and the mitotic timing measured. At least 70 cells per condition were analysed. Representative of 3 independent experiments. Please note that although cells expressing the YI/EQ mutant do not exit mitosis as fast as R262S, they still slip through the nocodazole arrest; by contrast most cells expressing wild type Cdc20 die during the arrest.

Supplemental Figure 3 - Related to Figure 4

(A) Structure of the ABBA motif from yeast Acm1 bound to the ABBA motif binding pocket in yeast Cdh1 labelled with the positions of the residues (Burton et al., 2011; He et al., 2013). (B) ABBA motif binding pocket residues of the yeast and human Cdc20-like proteins removed from a Clustal Omega alignment of the 32 CDC20-like proteins in twelve model eukaryotic organisms: Arabidopsis thaliana, Caenorhabditis elegans, Chlamydomonas reinhardtii, Dictyostelium discoideum, Drosophila melanogaster, Homo sapiens, Monosiga brevicollis, Phytophthora infestans (strain T30-4), Plasmodium falciparum (isolate 3D7), Saccharomyces cerevisiae (strain ATCC 204508 / S288c), Schizosaccharomyces pombe (strain 972 / ATCC 24843) and Xenopus tropicalis. (C) Relative binomial logo of the residues in the ABBA motif binding pocket from the 32 CDC20-like proteins. (D) Relative binomial logo created from the peptides within the Bub1/BubR1 alignment (see http://slim.ucd.ie/abbakenabba/ for alignments) aligned to the experimentally characterised instances of the GLEB motif core (the motif has extensions but they are not at a constant spacing) (Larsen et al., 2007). Aligned GLEBS motif peptides in the Bub1/BubR1 alignment: VGEFSFEEIRAEV, VGEFQPEEILAAC, RLEFSLEEVLAIS, RDDACYEERRAVR, QFELTLEELIARR, NMDQSMEEVRAQV, NIEFSPEEYRAYS,GVEYSPEEILARK, GEERSMEECRAQR, GEAISFEESRARA, ESEFSFEELRAQK, EEDCTFEELRANM, DHEFCIEEILALA, DEEFNTEEILAMI. (E) PSI-BLAST PSSM (position specific scoring matrix) of the core GLEBS motif peptides (as defined in panel D). PSSM created using the PSI-BLAST PSSM creation protocol including sequence weighting and pseudocounts (Altschul and Koonin, 1998; Schäffer et al., 2001). PSSM significance cut-off with p-value of 0.00001 calculated based on peptide randomisation. Bub1/BubR1 proteins were scanned with PSSM and a GLEBS motif is defined as present if a peptide scores above the PSSM significance cut-off. (F) Relative binomial logo created from the peptides within the Bub1/BubR1 alignment (See http://slim.ucd.ie/abbakenabba/_for alignments) aligned to the experimentally characterised Mad1 binding region (London and Biggins, 2014). Aligned Mad1 binding region peptides in the Bub1/BubR1 alignment: VQPSPTVHTKEALGFIMNMFQAP, RSPTVTMFSRDAMKEVYSMFNQH, RSPTVTAFSKDAINEVFSMFNQH, QDPTMTICTKEAWGDIMSMFSGG, NCPSPTINTKAAMADIMAMFGAP, LRMSPTLVTKAAVLEVENMFNGD, LGQSLTVNTKEAMSVVQDMWRSP, KPSSPTVCTKEAMGEIFGMFQKP, KPPSPTIHTKAALADILDIFNQP, GLVDPTVNLKEAMEDINNMFGEP, EQEDMTINTRVALEDINNMFCSP. (G) PSI-BLAST PSSM (position specific scoring matrix) of the Mad1 binding region peptides (as defined in panel F). PSSM

created as described in panel D. PSSM significance cut-off with p-value of 0.00001 calculated based on peptide randomisation. Bub1/BubR1 proteins were scanned with PSSM and a Mad1 binding region is defined as present if a peptide scores above the PSSM significance cut-off.

Supplemental Table 1 – Related to Figure 4
Search criteria for domains shown in Figure 4 are detailed.

Supplemental Experimental Procedures

Plasmid generation

BubR1, Cdc20 and their mutants were cloned into a modified version of pcDNA5/FRT/TO (Invitrogen), which contained at the N-terminus either FLAG tag alone or linked to the mRuby or Cerulean fluorescent proteins. Mutants and siRNA-resistant constructs were generated using overlapping PCR. For the $ABBA^{272}$ mutant the third, fourth and sixth positions were replaced with alanine, and for the $ABBA^{340}$ motif mutant the first, third, fourth and sixth positions were replaced with alanine. To test the role of the spacing between the motifs in the cassette, residues 280-292 and 319-336 were deleted. In the rescue plasmids a repeated Gly-Gly-Thr linker was introduced to restore the distance but not the sequence between motifs. All plasmids were verified by sequencing. Details of cloning are available on request.

RNA interference

siRNA duplex against BubR1 (GAUGGUGAAUUGUGGAAUA, Dharmacon), Cdc20 (CGGAAGACCUGCCGUUACAUU, Dharmacon) and ON-TARGETplus non-targeting siRNA pool (Sigma) were transfected at a final concentration of 50nM with RNAimax (Invitrogen) according to the manufacturer's protocol. For immunoprecipitation experiments a single siRNA transfection was performed before a single thymidine block protocol. For siRNA rescue experiments, a double siRNA transfection/double thymidine synchronization protocol was followed, with the first siRNA transfection performed before the first thymidine addition and the second siRNA transfection about 6 hours after release from the first thymidine block.

Immunoblotting

After electrophoresis on 4-12% gradient Bis-Tris acrylamide gels, proteins were transferred to a PVDF membrane. Membrane saturation and all the following incubation steps were carried out in 5% low fat milk in PBS-Tween 0.1%. The following primary antibodies were used at the indicated dilutions: FLAG (M2, Sigma, 1:1000), Mad2 (Bethyl Laboratories, 1:1000), actin (AC-15, Sigma, 1:1000), Cdc20 (Bethyl Laboratories, 1:500), APC4 (monoclonal antibody raised against a C-terminal peptide, 1:500), BubR1 (Bethyl Laboratories1:500), Bub3 (Bethyl Laboratories, 1:500), Bub1 (Abcam, 1:3000). IRDye680 and IRDye800CW (LI-COR)-conjugated secondary antibodies were used at 1:10000. The antibody signal was detected using the Odyssey infrared imaging system (LI-COR) for quantitative

immunoblotting.

Relative binomial logo construction

Relative binomial logos were calculated as $prob^{aa} = binomial(k,n,p)$ where k is the observed residue count at each position for a residue, n is the number of the instances of motifs and p is the background frequency of the residue in the intrinsically disordered regions of the human proteome. The grey line annotated as p(0.05) signifies the height of a amino acid that has a $prob^{aa}$ of 0.05. The data are split based on whether a residue is depleted or enriched at a particular position. A positive score (above the central partition containing the residue offset numbering) denotes that a residues is enriched, a negative score (below the partition) denotes that a residue is depleted, and score of zero suggests that a residue is occurring at the expected frequency.

Affinity and specificity determinants of the ABBA motif

List of experimentally characterised ABBA motif instances

Gene	Protein	Sequence	Reference
H. sapiens CDC20			
Bub1	Mitotic checkpoint kinase BUB1	slssa 527 **FHVFED** 532 gnken	(Di Fiore et al., 2015)
BubR1	Mitotic checkpoint kinase BUB1 beta	gpsvp 528 **FSIFDE** 533 fllse	(Di Fiore et al., 2015; Díaz-Martínez et al., 2015)
BubR1	Mitotic checkpoint kinase BUB1 beta	qnnsr 272 **ITVFDE** 277 nadea	*This study*
BubR1	Mitotic checkpoint kinase BUB1 beta	avlps 340 **FTPYVE** 345 etarq	*This study*
CCNA2	Cyclin-A2	skqpa 99 **FTIHVD** 104 eaeke	(Di Fiore et al., 2015)
S. cerevisiae Cdh1			
Acm1	APC/C-CDH1 modulator 1	skaaq 61 **FMLYEE** 66 taeer	(He et al., 2013) *{Burton:2011dl}*
S. cerevisiae Cdc20			
Clb5	S-phase entry cyclin-5	vsavq 99 **KRQIYND** 105 rtaae	(Lu et al., 2014)
Mad3	SAC component MAD3	dvnlt 258 **KNNVFVD** 264 geesd	*By homology* [a]
Mad3	SAC component MAD3	rpyde 330 **KLPIFRD** 336 sigrs	*By homology* [a]

[a] Mapped by homology from the human BubR1 ABBA motifs described in this study. The loss of the position +1 hydrophobic specificity and affinity determinant and the gain of a position -1 basic determinant observed and explained by Lu *et al* 2014 for Clb5 is once again observed in both Mad3 ABBA motifs.

Structural, sequence and evolutionary analysis of the available ABBA motif instances indicate that the metazoan Cdc20 and fungal Cdh1 ABBA motif binding pockets recognise peptides containing the consensus ϕ-x-ϕ-ϕ-x-D/E. In metazoa, the P1 position has been observed to allow a hydrophobic amino acid from the set of I/L/V/M/F. P3 position prefers an I/L/V, however the second human ABBA of the ABBA-KEN-ABBA cassette has a conservative substitution of another aliphatic residue – proline - in this position. The P4 position has a more constrained preference showing a strong enrichment for an aromatic residue, only a handful of instances diverge from this. As previously observed and explained by Lu *et al.,* 2014 for the Clb5 ABBA motif, the fungal Cdc20 ABBA motif binding pocket prefers K-x-x-ϕ-ϕ-x-D/E as a result of a change in the ABBA motif binding pocket.

Both ABBA motifs (mapped by homology) of the ABBA-KEN-ABBA cassette in yeast Mad3 match this preference.

The four original ABBA motifs in Cyclin A, Bub1, BubR1 and Acm1 indicated a consensus of Fx[ILV][FHY]x[DE]. This consensus is strongly conserved in all four instances but not in the ABBA-KEN-ABBA cassette where other hydrophobic residues are allowed, particularly in the P1 positions. The determinants may be less strictly required due to the multivalency of the interaction permitting a sub-optimal motif and may explain why the ABBA motifs of the ABBA-KEN-ABBA cassette are weaker binders than the original ABBA motifs: both contain an infrequently observed and potentially weaker amino acid at a single position: P1 for ITVFDE and P3 for FTPYVE.

Key residues in the potential ABBA binding pocket of human Cdh1, conserved across all metazoan Cdc20s and fungal Cdh1, strongly differ from the consensus – P338 and P355 (Figure S3A-B - yeast Cdh1 numbering). The replacement of the Arginine at P338 with a small amino acid, serine, would have a large effect on the binding pocket. Conversely, the ABBA binding pocket in yeast Cdh1 has been largely conserved relative to the human Cdc20 pocket, and the yeast Cdc20 binding pocket has diverged only in the ABBA motif P1 contacting positions. This would suggest that the metazoan Cdh1 ABBA binding pocket has had a large shift in specificity and no longer recognises "canonical" ABBA motifs, or, it no longer exists as a binding site for any motif. In agreement with this the human ABBA motifs BUB1 527-532, BUB1B 528-533 and Cyclin A 99-104 do not bind human Cdh (Di Fiore et al., 2015). .

Search criteria for homology mapping of functional modules in the Bub1-like protein family

The search criteria to define the conservation of the functional modules in the Bub1-like proteins used to construct figure 4C in the main text. See Table S1 for details of the homology-mapped modules.

Bub1-like proteins retrieved from 13 eukaryotic organisms were split into 3 groups: (i) single copy Bub1-like proteins; (ii) duplicated Bub1-like proteins; and (iii) duplicated BubR1-like proteins. Proteins from group (ii) and (iii) were aligned to the proteins from group (i) with the MSAProbs multiple sequence alignment tool to create two alignments (See http://slim.ucd.ie/abbakenabba/).

<u>*ABBA motif*</u>

Peptides from the Bub1/BubR1 alignment (See http://slim.ucd.ie/abbakenabba/) aligned to the experimentally characterised instances of the motif and matching the ϕxϕϕx[DE] or Kxxϕϕx[DE] consensus are defined as retained modules. Consensus definition is described above.

<u>*KEN box*</u>

Peptides from the Bub1/BubR1 alignment (See http://slim.ucd.ie/abbakenabba/) aligned to the experimentally characterised KEN box motif instances and matching the KEN consensus are defined as retained modules. Peptides from the alignment aligned to the experimentally characterised instances of the motif and matching the xEN consensus are defined as inconclusively retained modules.

Consensus derived from experimentally validated instances at the APC/C degron repository (http://slim.ucd.ie/apc/).

D box

Peptides from the Bub1/BubR1 alignment (See http://slim.ucd.ie/abbakenabba/) aligned to the experimentally characterised instances of the motif and matching the RxxLxx[ILMVK] consensus are defined as retained modules. Peptides aligned to the experimentally characterised instances of the motif and matching the RxxFxx[ILMVK] consensus are defined as inconclusively retained modules. Consensus derived from experimentally validated instances at the APC/C degron repository (http://slim.ucd.ie/apc/).

GLEBS

See Fig S3D-E

MAD1 binding region

See Fig S3F-G

TPR domain

Presence in the Bub1/BubR1 protein of an Interpro Mad3/Bub1 homology region 1 (IPR013212) domain

Kinase domain

Presence in the Bub1/BubR1 protein of an Interpro Protein kinase-like domain (IPR011009) domain

Supplemental References

Altschul, S.F., and Koonin, E.V. (1998). Iterated profile searches with PSI-BLAST--a tool for discovery in protein databases. Trends in Biochemical Sciences *23*, 444–447.

Burton, J.L., Xiong, Y., and Solomon, M.J. (2011). Mechanisms of pseudosubstrate inhibition of the anaphase promoting complex by Acm1. Embo J. *30*, 1818–1829.

Di Fiore, B., Davey, N.E., Hagting, A., Izawa, D., Mansfeld, J., Gibson, T.J., and Pines, J. (2015). The ABBA motif binds APC/C activators and is shared by APC/C substrates and regulators. Dev. Cell *32*, 358–372.

Díaz-Martínez, L.A., Tian, W., Li, B., Warrington, R., Jia, L., Brautigam, C.A., Luo, X., and Yu, H. (2015). The Cdc20-binding Phe box of the spindle checkpoint protein BubR1 maintains the mitotic checkpoint complex during mitosis. J. Biol. Chem. *290*, 2431–2443.

He, J., Chao, W.C.H., Zhang, Z., Yang, J., Cronin, N., and Barford, D. (2013). Insights into degron recognition by APC/C coactivators from the structure of an Acm1-Cdh1 complex. Mol. Cell *50*, 649–660.

Larsen, N.A., Al-Bassam, J., Wei, R.R., and Harrison, S.C. (2007). Structural analysis of Bub3 interactions in the mitotic spindle checkpoint. Proceedings of the National Academy of Sciences of the United States of America *104*, 1201–1206.

London, N., and Biggins, S. (2014). Mad1 kinetochore recruitment by Mps1-mediated phosphorylation of Bub1 signals the spindle checkpoint. Genes & Development *28*, 140–152.

Lu, D., Hsiao, J.Y., Davey, N.E., Van Voorhis, V.A., Foster, S.A., Tang, C., and Morgan, D.O. (2014). Multiple mechanisms determine the order of APC/C substrate degradation in mitosis. J. Cell Biol. *207*, 23–39.

Schäffer, A.A., Aravind, L., Madden, T.L., Shavirin, S., Spouge, J.L., Wolf, Y.I., Koonin, E.V., and Altschul, S.F. (2001). Improving the accuracy of PSI-BLAST protein database searches with composition-based statistics and other refinements. Nucleic Acids Research *29*, 2994–3005.
